# The Link between Morphotype Transition and Virulence in *Cryptococcus neoformans*

**DOI:** 10.1371/journal.ppat.1002765

**Published:** 2012-06-21

**Authors:** Linqi Wang, Bing Zhai, Xiaorong Lin

**Affiliations:** Department of Biology, Texas A&M University, College Station, Texas, United States of America; University of Birmingham, United Kingdom

## Abstract

*Cryptococcus neoformans* is a ubiquitous human fungal pathogen. This pathogen can undergo morphotype transition between the yeast and the filamentous form and such morphological transition has been implicated in virulence for decades. Morphotype transition is typically observed during mating, which is governed by pheromone signaling. Paradoxically, components specific to the pheromone signaling pathways play no or minimal direct roles in virulence. Thus, the link between morphotype transition and virulence and the underlying molecular mechanism remain elusive. Here, we demonstrate that filamentation can occur independent of pheromone signaling and mating, and both mating-dependent and mating-independent morphotype transition require the transcription factor Znf2. High expression of Znf2 is necessary and sufficient to initiate and maintain sex-independent filamentous growth under host-relevant conditions *in vitro* and during infection. Importantly, *ZNF2* overexpression abolishes fungal virulence in murine models of cryptococcosis. Thus, Znf2 bridges the sex-independent morphotype transition and fungal pathogenicity. The impacts of Znf2 on morphological switch and pathogenicity are at least partly mediated through its effects on cell adhesion property. Cfl1, a Znf2 downstream factor, regulates morphogenesis, cell adhesion, biofilm formation, and virulence. Cfl1 is the first adhesin discovered in the phylum Basidiomycota of the Kingdom Fungi. Together with previous findings in other eukaryotic pathogens, our findings support a convergent evolution of plasticity in morphology and its impact on cell adhesion as a critical adaptive trait for pathogenesis.

## Author Summary

Although morphogenesis and virulence are commonly associated in many eukaryotic pathogens, the nature of such association is often unknown. For example, *Cryptococcus neoformans*, a fungal pathogen that causes cryptococcal meningitis, typically undergoes morphological transition between the yeast and the filamentous form during mating. However, molecules that are critical for mating do not directly impact fungal virulence. Thus, the nature of the long observed association between morphotype and virulence in this microbe remains elusive despite decades of effort. Here we demonstrate that constitutively activated pheromone signaling is insufficient to drive morphological transition under mating-suppressing conditions, including those relevant to host physiology. Rather, we demonstrate that sex-independent morphological switching is driven by the transcription factor Znf2 and this regulator controls the ability of this fungus to cause disease. Znf2 governs *Cryptococcus* morphotype and virulence potential at least partly through its effects on cell surface proteins. One novel adhesin Cfl1functions downstream of Znf2 and it orchestrates morphological switch, cell adhesion, biofilm formation, and pathogenicity. Thus, cell adhesion at least partly underlies the link between morphological transition and pathogenicity in *C. neoformans*. Our findings provide a platform for further elucidation of the impact of morphotype on virulence in this ubiquitous pathogen. The discovery of Cfl1 and other novel adhesins in *Cryptococcus* could lay a foundation for the development of vaccines or alternative therapies to combat the fatal diseases caused by this fungus.

## Introduction

Adaptation to the host environment by many eukaryotic pathogens is often companied by transition in cellular morphology [1], [2], [3], [4], [5], [6], [7], [8], [9]. The ubiquitous fungal pathogen *Cryptococcus neoformans* causes more than half a million deaths each year [10]. It can grow in the yeast form as well as the filamentous form. Earlier pre-genetic studies indicate an inverse relationship between filamentation and virulence [11], [12], [13], [14], [15], [16], [17]. These studies also point to the potential of filament-specific antigens as vaccines against *Cryptococcus* infections [18], [19], [20].

Because *Cryptococcus* typically grows in the yeast form and the morphological transition from the yeast form to the filamentous form appears to be coupled with mating, signaling pathways that lead to bisexual mating (**a**-α mating) and unisexual mating (mostly α-α mating) have been intensively investigated [21], [22], [23], [24]. The roles of these signaling components in fungal pathogenicity are also scrutinized in animal models. However, accumulating evidence indicates that key signaling components that specifically lead to mating, such as those in the pheromone sensing pathway, have no or minimal direct effect on virulence [25], [26], [27], [28]. Furthermore, conditions relevant to host physiology (e.g. aqueous environment, high temperatures, and high levels of CO_2_) are mating-suppressive, suggesting sex-independent mechanisms in orchestrating morphotype and virulence in *Cryptococcus* [29]. Therefore, the existence and the nature of the link between morphological transition and virulence in *Cryptococcus* remain enigmatic.

## Results

### Activation of Pheromone Signaling Is Insufficient to Drive Filamentation under Mating-Suppressing Conditions

Although *Cryptococcus* morphological transition from the yeast form to the filamentous form is historically associated with mating, the observations that filamentation can be achieved in strains in the absence of key pheromone signaling components or meiotic genes [30], [31], [32], [33], lead us to hypothesize that pheromone signaling pathways are not essential or sufficient for filamentation *per se*, but they are critical in stimulating filamentation in response to mating cues. To test this hypothesis, we decided to examine the effect of constitutive activation of the pheromone signaling circuit on morphogenesis under mating-inducing and mating-suppressing conditions.

**Figure 1:**
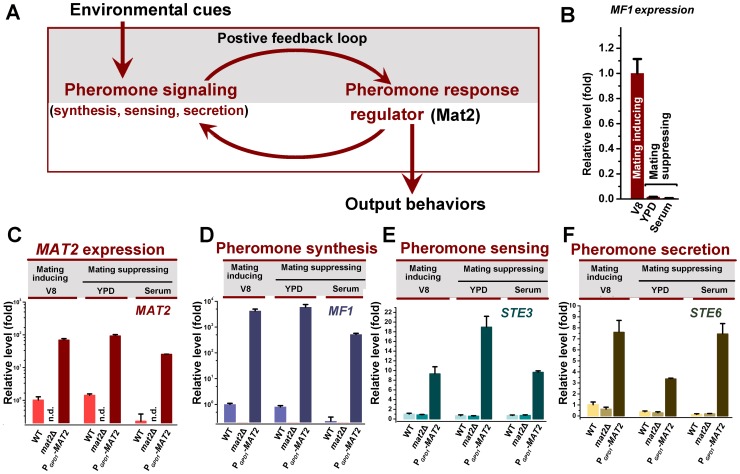
Overexpression of *MAT2* causes constitutive activation of pheromone signaling. (A) The *C. neoformans* pheromone signaling pathway. Pheromone signaling is triggered by environmental cues (mating cues) and it turns on the master regulator Mat2, which in turn activates pheromone signaling, thereby constituting a self-reinforcing system. Activated pheromone signaling determines the output mating-relevant behaviors (e.g. formation of shmoo cells and mating projections, and initiation and cell contact and cell fusion). (B) *MF1*α (pheromone) and other mating signal genes (not shown here) were highly induced in **a** x α cocultures under the mating-inducing condition (V8), but not under mating-suppressing conditions (YPD and Serum). Because unisexual mating is not observed in the wildtype strain, the induction of pheromone was evaluated during bisexual mating with the coculture of H99α and its congenic partner KN99**a** incubated on different media for 72 hr. The expression level of *MF1*α during bisexual mating on V8 medium was arbitrarily set as 1 for comparison. (C, D, E and F) In order to analyze the effect of the deletion or the overexpression of *MAT2* on the key elements of the pheromone pathway and to avoid potential complication due to higher expression of these elements in the presence of a compatible mating partner under mating-inducing conditions, only α strains alone in the H99 background were used in the these assays. Overexpression of *MAT2* constitutively activated pheromone signaling in single strain cultures under all the conditions tested. The expression patterns of *MAT2* (C), *MF1*α (D), *STE3* (pheromone receptor gene) (E), and *STE6* (pheromone transporter gene) (F) in wildtype (H99), and its derived *mat2*Δ mutant and the P*_GPD1_-MAT2* strain were shown. Gene expression levels in the wildtype H99 grown on V8 medium were arbitrarily set as 1 for comparison. Cells were cultured on different media for 72 hr.

**Figure 2:**
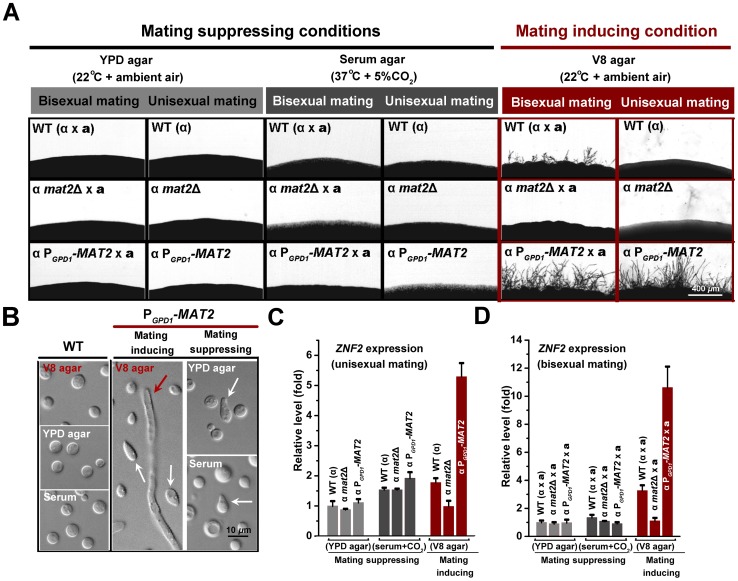
Activated pheromone signaling triggers formation of shmoo cells or mating projections, but not filaments under mating-suppressing conditions. (A) Overexpression of *MAT2* drove filamentation during unisexual mating (α cell culture alone) or bilateral mating (**a**-α cocultures) only under mating-inducing condition. (B) The pheromone overexpression strain P*_GPD1_-MAT2* was able to form shmoo-like cells irrespective of culture conditions. White arrows indicate shmoo-like cells and the red arrow indicates a hypha cell. (C and D) Activated pheromone signaling only induced the transcription of *ZNF2* under mating-inducing condition either during unisexual mating (α cell culture alone) (C) or during bisexual mating (**a**-α cocultures) (D). Transcript levels of *ZNF2* in the wildtype H99 grown on YPD agar were arbitrarily set as 1 for comparison. The cells were cultured on different media for 72 hr.

It is known that the expression of genes in the pheromone signaling pathway, such as those encoding the pheromone Mf1α, the pheromone receptor Ste3α, the pheromone transporter Ste6, and the key pheromone response regulator Mat2 (Figure 1A), is low under mating-suppressing conditions but is dramatically higher during **a**-α bisexual mating (Figure 1B and data not shown) [30], [32]. We found that the expression level of these pheromone signaling genes in wildtype α strain H99 alone was low when cells were cultured on either mating-inducing condition (V8 agar) or mating-suppressing conditions (YPD agar and serum) (Figure 1B, C, D, E and F). This is consistent with the well-noted poor ability of the H99 strain to undergo unisexual mating. In fact, filamentation has never been observed when H99 was cultured alone under mating-inducing conditions (Figure 2A) [34]. We chose this strain to study the link between morphogenesis and virulence because H99 is one of the most virulent clinical strains tested in various animal models and it is also widely used as a reference strain in *Cryptococcus* research.

When we placed the *MAT2* gene under the control of the constitutively active promoter of *GPD1* (glycerol-3-phosphate dehydrogenase 1) and introduced this construct to H99, the transcript level of *MAT2* was dramatically increased under mating-inducing as well as mating-suppressing conditions (Figure 1C). As expected for a key regulator of the pheromone signaling, overexpression of *MAT2* led to high expression of *MF1*α, *STE3*α, and *STE6* under both mating-inducing and mating-suppressing conditions (Figure 1D, E and F). This result indicates that constitutively overexpression of *MAT*2 is sufficient to induce pheromone signaling circuit independent of mating cues.

We next tested the effect of activation of pheromone signaling on filamentation under different conditions. The P*_GPD1_-MAT2* conferred filamentation to H99 during unisexual mating (α cells alone) and it significantly enhanced filamentation during bisexual mating (**a**-α coculture) under the mating-inducing condition (Figure 2A). However, under mating-suppressing conditions, overexpression of *MAT2* failed to stimulate filamentation in the α alone culture or in the **a**-α coculture (Figure 2A), and this was not due to insufficient activation of pheromone signaling. Effective activation of pheromone signaling in the P*_GPD1_-MAT2* strain is supported by both the high expression levels of genes involved in pheromone signaling (Figure 1D, 1E, and 1F) and the formation of shmoo-like cells under both mating-inducing and mating-suppressing conditions (Figure 2B). Shmoo-like cells are typically observed when cells respond to mating signals prior to cell fusion. These observations indicate that activation of pheromone signaling alone is not sufficient to initiate filamentation under mating-suppressive conditions, including conditions relevant to host physiology. Thus mating signaling is unable to coordinate the yeast-filament morphological transition and virulence during infections.

### Filamentation Can Be Independent of Sex and Is Controlled by the Transcription Factor Znf2

We previously showed that the deletion of *ZNF2*, which encodes a zinc-finger transcription factor, locked cells in the yeast form during mating without impairing pheromone signaling [28]. This suggests that Znf2 is not essential for mating signal relay; rather, it is crucial for filamentation. Although Znf2 functions downstream of Mat2 during mating [28] and its gene expression was significantly induced by *MAT2* overexpression under the mating-inducing condition (Figure 2C and 2D), activation of the pheromone signaling pathway was unable to induce *ZNF2* expression in the absence of mating stimuli. This was evidenced by the low expression level of *ZNF2* in the P*_GPD1_-MAT2* strain under mating-suppressing conditions (Figure 2C and 2D). The ability of *Cryptococcus* to undergo filamentation correlates with the expression level of *ZNF2*, but not that of *MAT2* (Figure 1C, Figure 2A, 2C and 2D). Thus, Znf2 could be a master regulator that dictates *Cryptococcus* morphotype irrespective of environmental stimuli or mating type.

**Figure 3:**
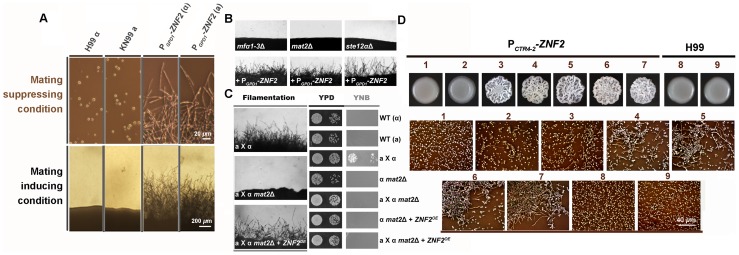
Znf2 is a master regulator of filamentation. (A) Wildtype congenic pair H99α and KN99a, and their derived P*_GPD1_-ZNF2* strains were grown at 22°C on V8 juice agar medium (mating-inducing condition, scale bar: 200 µm) for 5 days or in YPD liquid medium (mating-suppressing condition, scale bar: 20 µm) (see Figure S1 for images of strains in serotype D backgrounds). (B) The *mf*α*1-3*Δ, *mat2*Δ, and *ste12*αΔ mutants in the JEC21 background and their transformants with the P*_GPD1_*-*ZNF2* construct were incubated on V8 agar medium for 2 days. Wildtype JEC21 can self-filament sporadically and poorly only after prolonged incubation (>1 week) under this condition. (C) For cell fusion assays, auxotrophic parental cells of either a or α mating type were cultured alone or together on V8 agar medium for 24 hrs. Cells were then collected, washed, and spotted onto rich YPD medium for growth control or onto minimal YNB medium to select prototrophic cell-fusion products. The images of cocultures on V8 agar medium are shown to the left. See Figure S2 and 3 for the effect of Znf2 and Mat2 on hyphal maintenance under host conditions, as well as expression of genes related to cell fusion and mating projection formation during mating. (D) The P*_CTR4-2_-ZNF2* strain and the wildtype H99 were incubated on YPD media that contain either BCS (inducer) or CuSO_4_ (inhibitor) of varied concentrations. Cells scraped from the colony above were examined microscopically (shown below). 1: 25 µM CuSO_4_, 2: 0 CuSO_4_, 3: 5 µM BCS, 4: 25 µM BCS, 5: 50 µM BCS, 6: 100 µM BCS, 7: 200 µM BCS, 4, 8: 25 µM CuSO_4_, and 9: 200 µM BCS.

To test this hypothesis, we constructed the P*_GPD1_-ZNF2* strains. Indeed, the P*_GPD1_-ZNF2* triggered filamentation in *Cryptococcus* strains of either mating types **a** or α in both serotype A and serotype D backgrounds under all tested conditions, including those that are inducing or suppressive to mating (Figure 3A and Figure S1). In contrast to the P*_GPD1_-MAT2* strain, filaments produced by the P*_GPD1_-ZNF2* strain under mating-inducing condition maintained their filamentous morphology after being transferred to mating-suppressive conditions (Figure S2). However, it is notable that the P*_GPD1_*-*ZNF2* strain produces more robust hyphae under mating-inducing condition, suggesting that other factors induced under mating-inducing condition could further activate Znf2.

The P*_GPD1_-ZNF2* also conferred filamentation to mutants that harbor deletions in the key mating components under various conditions tested (Mfα1-3, Mat2, or Ste12 functioning in a branching pathway in pheromone signaling) (Figure 3B). To confirm that filamentation conferred by Znf2 activation is not due to some cryptic restoration of mating ability, we measured the efficiency of cell fusion of the wildtype, the *mat2*Δ mutant, and the *mat2*Δ+P*_GPD1_-ZNF2* strain during bisexual **a**-α mating. Indeed, overexpression of *ZNF2* did not rescue the cell fusion defects of the *mat2*Δ mutant (Figure 3C). Consistently, gene ontology analyses of our previous transcription data indicate that Znf2, unlike Mat2, does not regulate genes involved in the cell fusion event critical for mating (Figure S3) [28]. Taken together, the results indicate that filamentation can be independent of mating and Znf2 is one key determinant of this sex-independent morphogenesis.

### Expression Level of Znf2 Mediates Bi-directional Morphological Transition

To verify the correlation of *ZNF2* expression and *Cryptococcus* morphology, we constructed the *ZNF2* gene driven by two inducible promoters: the galactose-inducible *GAL10* promoter (data not shown) [35] or the copper transporter *CTR4* promoter (Figure 3D) (copper deprivation–on; copper repletion–off) [36]. Transformation of the P*_GAL10_-ZNF2* or the P*_CTR4-2_-ZNF2* construct into wildtype either the serotype D reference strain JEC21 or the serotype A reference strain H99 conferred filamentous growth under promoter-inducing conditions. These strains grew as yeasts under promoter-repressive conditions (Figure 3D and data not shown). Increasing the concentration of the copper chelator BCS (inducer) increased the frequency of filamentation in the P*_CTR4-2_-ZNF2* strain (Figure 3D), indicating that the expression level of *ZNF2* dictates *Cryptococcus* cellular morphology.

**Figure 4:**
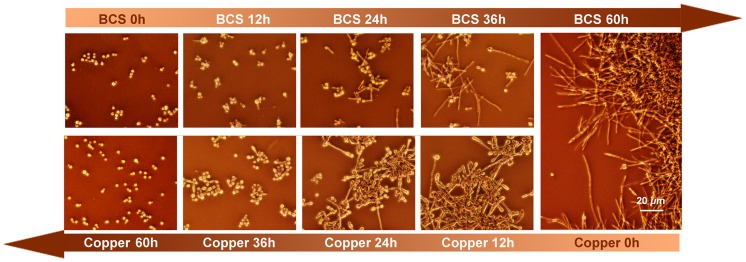
Znf2 governs bi-directional morphological transition and morphotype maintenance. Yeast cells of the P*_CTR4-2_-ZNF2* strain were incubated in YPD liquid medium supplemented with 200 µM BCS at 30°C. At 60 hrs, the filaments were collected, washed, and transferred to fresh YPD liquid medium containing 25 µM CuSO_4_. The wildtype strain remained in the yeast form under such conditions. Images were taken at indicated time points.

To examine the effect of *ZNF2* on the dynamic morphological transition, we incubated the P*_CTR4-2_* -*ZNF2* strain in H99 background in liquid YPD medium containing 200 µM BCS (inducer) and examined cell morphology over time. Morphological transition from the yeast form to the filamentous form completed by 60 hours (Figure 4). At this time, the hyphae were transferred to YPD medium containing copper sulfate (inhibitor). *Cryptococcus* cells then switched from the filamentous form to the yeast form over time (Figure 4). The control of bi-directional morphological transition by Znf2 is also observed when cells were cultured in serum (data not shown), indicating that this control is independent of environmental cues. These results demonstrate that (i) the expression level of *ZNF2* determines *Cryptococcus* cell morphology: high expression level of *ZNF2* drives the cells to the filamentous form and low expression level of *ZNF2* renders cells unicellular yeast; (ii) Znf2 is necessary and sufficient to initiate morphological transition; (iii) High Znf2 activity is required to maintain cells in the filamentous morphotype.

### Znf2 Controls Fungal Ability to Cause Disease

The relationship between morphotype and pathogenicity is typically defined through studying morphological mutants that are otherwise isogenic to the wildtype strains and are able to maintain given morphotype under host relevant conditions, even though mutants with such extreme phenotypes are unlikely to be encountered clinically due to natural selections in the host [1], [2], [3], [4]. For *Cryptococcus*, host physiological environment (e.g. high body temperature, aqueous environment, and high levels of CO_2_) is extremely inhibitory to mating. Consistently, constitutively activated mating signaling induced filamentation under mating-inducing conditions, such filaments could not be maintained when transferred to *in vitro* conditions that mimicked host physiological environment (Figure S2). In contrast, the P*_GPD1_-ZNF2* strain can readily initiate and maintain filamentous growth under such host-relevant conditions (Figure S2). Thus *ZNF2* overexpression strains could serve as a model to investigate the relationship between morphotype and pathogenicity.

**Figure 5:**
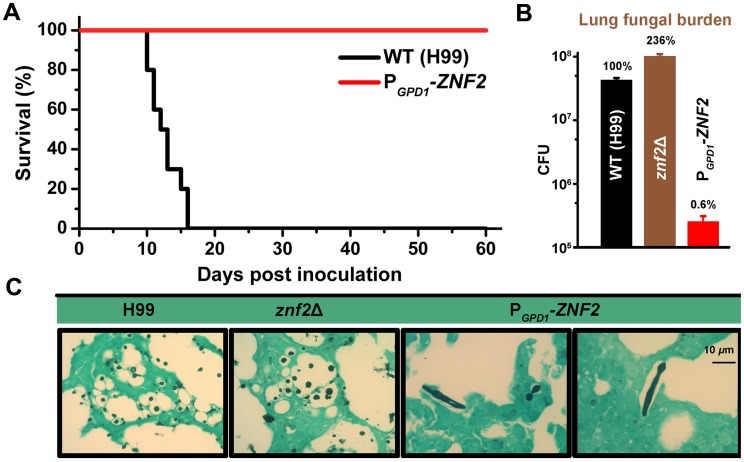
Znf2 links morphogenesis and pathogenicity. (A) Mice were infected intranasally with either the wildtype H99 or the P*_GPD1_-ZNF2* strain. Survival rate was plotted against days after inoculation. (B) Mice were infected with the wildtype H99, the *znf2*Δ mutant, and the P*_GPD1_-ZNF2* strain. Fungal burden in the lungs and the brains (Figure S4) was determined at DPI 10. Differences among the groups are statistically significant. (C) Lung tissues were stained with the Grocott Methenamine Silver stain. Fungal cells appear black or dark brown. Only tissues at DPI 12 are shown here. See Figure S4 for the correlation between brain fungal burden and Znf2 activity; see Figure S5 for the fungal cell morphology during the course of infection.

We tested the virulence of the wildtype H99 and the P*_GPD1_*-*ZNF2* strain in the murine inhalation model of cryptococcosis. The P*_GPD1_*-*ZNF2* strain exhibited heterogeneity in cell morphology and a mixture of cell types is always present in this strain. To obtain accurate inoculation and to avoid potential problems caused by differences in cell types at initial infection, only cells in the yeast form were used for animal inoculation. Remarkably, the P*_GPD1_*-*ZNF2* strain was completely avirulent (Figure 5A). By day 60 post infection (DPI 60) when the study was terminated, the P*_GPD1_*-*ZNF2* cells were either completely cleared from animal lungs or existed in very low numbers (1000 fold lower than the original inocula). We further examined the fungal burden in the lungs and the brain of animals infected with H99, the *znf2*Δ mutant, and the P*_GPD1_*-*ZNF2* strain at DPI 10 before any animal succumbed to cryptococcosis. Consistent with the animal survival rates, the lung fungal burden in animals infected with the *znf2*Δ mutant and the P*_GPD1_*-*ZNF2* strain was 236% and 0.6% respectively compared to those infected with the wildtype (Figure 5B). The brain fungal burden showed a similar trend with larger variations due to individual differences in the timing of dissemination in this inhalation model (Figure S4), and no fungal cells were recovered from the brains of animals infected by the P*_GPD1_*-*ZNF2* strain. To examine the effects of Znf2 on fungal morphology *in vivo*, we infected animals intranasally with H99, the *znf2*Δ mutant, and the P*_GPD1_*-*ZNF2* strain and performed histological examination of lung tissues at DPI 1, 7, and 12. Remarkably, even though only yeast cells from the P*_GPD1_*-*ZNF2* strain were used in the original inoculation into animals, lungs infected by the P*_GPD1_*-*ZNF2* strain contained *Cryptococcus* cells of mixed morphology: yeast, pseudohyphae, and hyphae in all the time points examined (Figure 5C and Figure S5). This is consistent with the morphological heterogeneity of the P*_GPD1_*-*ZNF2* strain *in vitro* (Figure S2). In comparison, only yeast cells were observed in the wildtype H99 or the *znf2*Δ mutant infected animals (Figure 5C and Figure S5). This histological examination indicates that activation of Znf2 can drive filamentation *in vivo*.

### Znf2 Controls Cell Adhesion through Its Regulation of Adhesion Proteins

Tolerance of host temperatures is a pre-requisite of fungal virulence. In some fungal pathogens, morphological changes are often a response to temperature and some morphological defective mutants lose the ability to cause diseases in mammalian hosts due to growth inhibition by high temperatures *in vivo*. To determine if alteration of virulence potential in the *znf2* mutants are simply due to altered sensitivity to high temperature, we compared the growth of the wildtype H99, the *znf2*Δ mutant, and the P*_CTR4-2_-ZNF2* strain at 30°C and 37°C on a variety of media *via* the spot assay. No apparent growth defects were observed in the *znf2*Δ mutant or the *ZNF2* overexpression strain when compared to the wildtype under the conditions tested (Figure S6). Furthermore, the observation that the *ZNF2* overexpression strain was capable of amplification during early stages of infection based on the fungal burden time course experiment (Figure S7) also suggests that factors other than growth inhibition by high temperature are mainly responsible for the effects of Znf2 on virulence.

**Figure 6:**
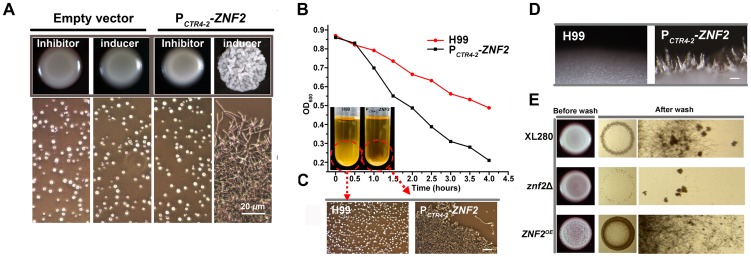
Znf2 regulates cell adhesion in *Cryptococcus*. (A) The P*_CTR4-2_-ZNF2* strain and a strain transformed with the empty vector (control) were incubated on YPD agar medium that contains either BCS (inducer) or CuSO_4_ (inhibitor). Cells scraped from the colony were examined microscopically (images below). (B) Wildtype H99 and the P*_CTR4-2_-ZNF2* strain were pre-grown in YPD medium containing 25 µM CuSO_4_ (inhibitor) for 12 hrs (no cell aggregation). The yeast cells were washed twice, inoculated into fresh YPD medium containing 200 µM BCS (inducer) and grown for additional 4 hrs with shaking before they were allowed to settle. Cell concentration of the upper stagnate culture (OD_600_) was measured every 30 min. (C) Cells from the bottom of the cultures were examined microscopically. (D) Overexpression of *ZNF2* leads to the formation of hyphal bundles. Wildtype H99 and its derived P*_CTR4-2_-ZNF2* strain were grown on YPD BCS agar plate at 22°C for 5 days. Multiple hyphae were attached together forming bundles in the P*_CTR4-2_*-*ZNF2* strain (scale bar: 100 µm). (E) Znf2 controls invasive growth. Wildtype XL280 and its derived *znf2*Δ mutant and the P*_GPD1_-ZNF2* strain were grown on YPD agar medium at 22°C for 5 days. The left column shows the original colonies; the middle column shows invasive cells after surface cells were washed off; and the right column shows enlarged images of the remaining invasive cells.

As morphological changes reflect changes in cell surface properties, we predict that Znf2 controls cell surface constitutes. One property likely regulated by Znf2 is cell adhesion, as supported by the following observations. First, increasing the *ZNF2* expression led to increasingly wrinkled colony morphology and flocculation (Figure 3D, and Figure 6A, B and C). Both phenotypes are likely caused by increased expression of flocculins (adhesins or adhesion proteins), as previously shown in bacteria and in yeasts [37], [38]. Second, aerial hyphae of the *ZNF2* overexpression strains formed on solid media also tended to attach to each other, forming bundles (Figure 6D), as observed in flocculated strains of the filamentous fungus *Ashbya gossypii* [39]. Third, deletion of *ZNF2* impairs agar invasion whereas overexpression of *ZNF2* remarkably promoted invasive growth (Figure 6E), and invasive growth reflects cell-substrate adhesion. The results suggest that Znf2 plays a pivotal role in morphogenesis-associated cell flocculation in *Cryptococcus*.

**Figure 7:**
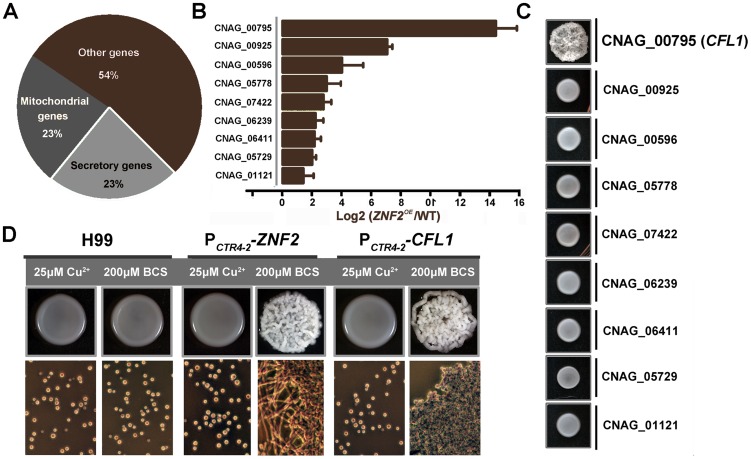
Znf2 regulates the expression of many extracellular Proteins. (A) Classification of genes differentially expressed in *znf2*Δ mutants compared with wildtype. (B) Selected genes predicted to encode extracellular proteins were also differentially expressed in the *ZNF2* overexpression strains by qPCR. (C) Gene overexpression strains in the H99 background were grown on YPD medium at 22°C for 3 days. (D) H99, the P*_CTR4-2_-ZNF2* strain, and the P*_CTR4-2_-CFL1* strain were pre-grown for 12 hrs in YPD liquid medium containing CuSO_4_ (inhibitor). The yeast cells were washed twice and then incubated on YPD agar medium containing BCS (inducer) for 3 days. Cells scraped from the colony above were examined microscopically (shown below).

Given that *Cryptococcus* strains with increased flocculation are reduced in virulence [40], [41], this transcription factor likely impacts pathogenicity at least partly through its effects on cell adhesion. Ontology analysis of our previous transcriptional profiling data [42] revealed that of those genes that are differentially expressed in the *znf2*Δ mutants, 23% encode secretory proteins based on WoLF PSORT prediction (http://wolfpsort.org/) (Figure 7A). We selected 9 such genes and examined their transcript level in a *ZNF2* overexpression strain incubated in serum at 37°C in 5% CO_2_ by quantitative realtime PCR. All genes tested were also differentially expressed in the *ZNF2* overexpression strain (Figure 7B).

We overexpressed these 9 genes using the constitutively active *GPD1* promoter and examined if their overexpression could recapitulate some of the phenotypes caused by the *ZNF2* overexpression (Figure 7C). Interestingly, strains with overexpression of CNAG_00795 (designated as *CFL1*: Cell FLocculin 1) formed extremely wrinkled colonies, like *ZNF2* overexpression strains (Figure 6A). Interestingly, the expression of *CFL1* was also most dramatically induced by the *ZNF2* overexpression (Figure 7B). Because acapsular *Cryptococcus* mutants also form wrinkled colony, we examined capsule production in the *CFL1* overexpression strain and *cfl1*Δ mutants. No apparent defect in capsule production was detected based on microscopic examination (data not shown).

To confirm that cell adhesion is indeed caused by increased *CFL1* expression, we then constructed P*_CTR4-2_*-*CFL1* strains. These strains grew as yeast cells in liquid cultures. A sharp increase in cell aggregation was observed when P*_CTR4-2_*-*CFL1* cells were cultured under promoter-inducing conditions, a reminiscence of some of the phenotypes of the P*_CTR4-2_*-*ZNF2* strains (Figure 7D).

**Figure 8:**
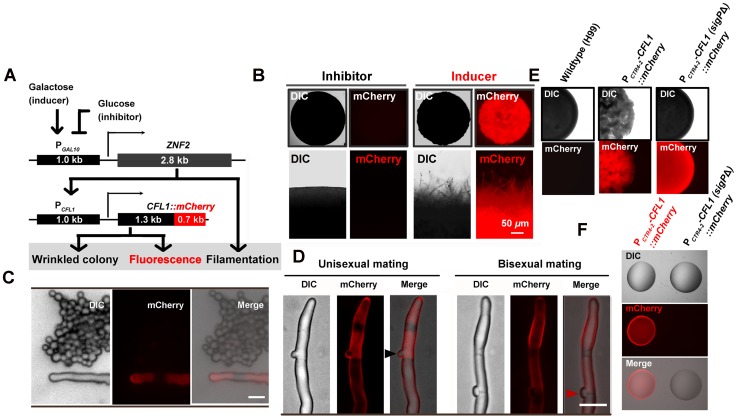
Cfl1 is a hypha-specific adhesin regulated by Znf2. (A) Diagram of the reporter system. The *znf2*Δ mutant carries both the P*_GAL10_-ZNF2* construct (*ZNF2* driven by the galactose-inducible promoter) and the P*_CFL1_-CFL1-m-Cherry* construct (fluorescent Cfl1 driven by its native promoter). The output behaviors (fluorescence, wrinkled colony morphology, and filamentation) are determined by the input signals (inducer/galactose or inhibitor/glucose). (B) The reporter strain showed wrinkled colony morphology and filamentation under the inducing condition (galactose). Under the suppressing condition (glucose), only smooth yeast colony without fluorescent was observed. (C and D) Cfl1-mCherry under the control of *CFL1* native promoter was detected in hyphae but not in yeasts. It was detected during both unisexual mating (characteristic unfused clamp cells, pointed by the black arrow) and bisexual mating (characteristic fused clamp cells, pointed by red arrow). Scale bars: 10 µm. (E) The coding region of the predicted N-terminal signal peptide of *CFL1* was deleted in frame in the construct of P*_CTR4-2_-CFL1(sigP*Δ*)-mCherry*. Both the P*_CTR4-2_-CFL1-mCherry* strain and the P*_CTR4-2_-CFL1(sigP*Δ*)-mCherry* strain showed cherry fluorescence under the inducing condition in the presence of BCS. Wrinkled colony morphology was observed in the P*_CTR4-2_-CFL1-mCherry* strain but not in the P*_CTR4-2_-CFL1(sigP*Δ*)-mCherry* strain. (F) The P*_CTR4-2_-CFL1-mCherry* strain and the P*_CTR4-2_-CFL1(sigP*Δ*)-mCherry* strain were grown in YPD liquid medium at 22°C for 7 days. The cultures with the same cell density were centrifuged and the supernatants were collected and filtered to remove the residual cells. Two microliters of the corresponding culture supernatants were spotted onto a glass slide and observed microscopically.

To further confirm that *CFL1* is regulated by Znf2, we engineered a reporter strain where *ZNF2* expression is inducible by galactose and the fluorescent Cfl1 is driven by its native promoter. We grew the reporter strain under mating-suppressing conditions to avoid complication due to potential activation of mating signaling. Under such conditions, the colony formed by the reporter strain became fluorescent and wrinkled when the *ZNF2* expression was induced in the presence of galactose (Figure 8A and B), while the colony was non-fluorescent and smooth when the *ZNF2* expression was inhibited in the presence of glucose (Figure 8A and B). Thus the expression of the fluorescent Cfl1 is driven by Znf2. Taken together, Znf2 triggers morphological switch as well as flocculation (cell adhesion), and its downstream factor Cfl1 regulates cell adhesion.

### Cfl1 Is Morphotype-specific and Its Secretion Is Required for Cell Adhesion

We examined the sub-localization of Cfl1 using a strain harboring the mCherry fused Cfl1 protein driven by its native promoter. Because Cfl1 is induced during mating and controlled by key components of mating signaling (Figure S8A and B), we examined microscopically the expression of *CFL1*-m-cherry during mating. Cfl1 was rarely detected in yeast cells (Figure 8C), but it was highly expressed in hyphae during both unisexual mating and bisexual mating (Figure 8D). The fluorescent Cfl1 delineated the periphery of hyphal cells, consistent with the function of adhesins on the cell surface and the prediction that Cfl1 is a secretory protein based on the presence of an N-terminal signal peptide for secretion.

Secretion is required for Cfl1's function as an adhesin. This is supported by the observation that overexpression of the fluorescent Cfl1 that lacks the N-terminal signal peptide [Cfl1(sigPΔ)-mCherry] failed to confer wrinkled colony morphology or cell aggregation to *Cryptococcus* (Figure S9 and Figure 8E). This is not due to a failure of producing the mutant allele protein, as abundant Cfl1(sigPΔ)-mCherry protein was produced by the cells (Figure 8E). However, no fluorescence was detected from the culture supernatant (Figure 8F), indicating defects in secretion. A few other fungal adhesins are also reported to be associated with cell surface as well as being released into surrounding environment [43], [44]. Such property may facilitate their roles in mediating both cell-cell adhesion and cell-substrate adhesion, and it may also help circumvent the blockage by other extracellular components. Consistent with its role as an adhesin, Cfl1 regulates a broad spectrum of cell adhesion-related biological processes, including complex colony morphology [45], [46] and formation of different biofilms (Figure S10).

**Figure 9:**
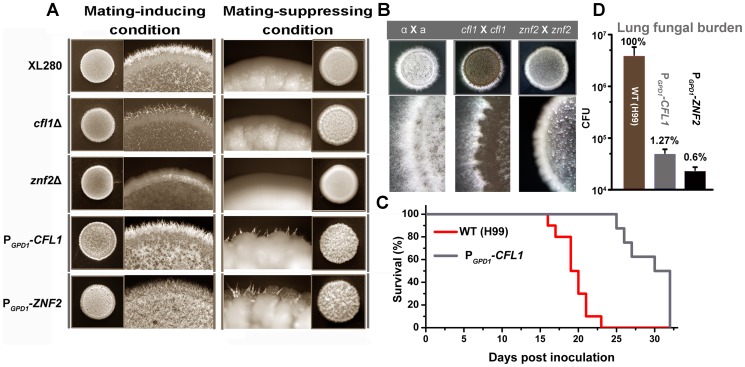
Cfl1 plays important roles in hyphal morphogenesis and pathogenicity in *Cryptococcus*. (A) Cfl1 affects hyphal formation in the hyper-filamentous wildtype strain XL280 under both mating-inducing and mating-suppressing conditions. (B) Deletion of *CFL1* reduced filamentation during bisexual mating. (C and D) Mice were infected intranasally with either wildtype H99 or the P*_GPD1_-CFL1* strain. Survival rate was plotted against days after inoculation. Fungal burden in the lungs at DPI 7 was shown. Differences among the groups are statistically significant (*p*<0.05).

Remarkably, deletion of *CFL1* dramatically reduced hyphal production during either bisexual or unisexual mating while overexpression of *CFL1* enhanced the hyphal formation (Figure 9A and B). Thus, both the expression pattern of *CFL1* and the observed effects of *CFL1* deletion or overexpression on hyphal development indicate the importance of this adhesin in hyphal morphogenesis. Like the *ZNF2* overexpression strain, hyphae formed by the P*_GPD1_-CFL1* strain on YPD medium (mating-suppressive) tended to attach to each other, forming bundles (Figure 6C and Figure 9A).

### Cfl1 Affects Fungal Virulence

Previous studies implicate an inverse association between flocculation and virulence in *Cryptococcus* [40], [41]. Consistently, we found that overexpression of *CFL1* resulted in attenuation in virulence, indicating that Cfl1-mediated cell adhesion negatively modulates virulence (Figure 9C). Consistently, organ fungal burdens were maintained at low level in the P*_GPD1_-CFL1* and P*_GPD1_-ZNF2* infected animals at DPI 7, whereas the wildtype H99 strain proliferated significantly (Figure 9D). Unlike the P*_GPD1_-ZNF2* strain, the P*_GPD1_-CFL1* strain was not completely avirulent and the P*_GPD1_-CFL1* strain proliferated significantly when examined at DPI 12 (Figure S11). This is surprising but not unexpected as the impact of *ZNF2* overexpression is likely the combinational effect of additional adhesion proteins and morphogenesis factors. As noted for *znf2* mutations, deletion or overexpression of *CFL1* did not cause any apparent change in growth compared to wildtype when cultured at 37°C with 5% CO_2_ (Figure S12A and B). Cells aggregated when *CFL1* was overexpressed at both 30°C and 37°C as expected.

## Discussion

*C. neoformans* is the major fungal pathogen from the phylum Basidiomycota in the Kingdom Fungi. Its morphological differentiation is typically heterogeneous and stochastic, and has been historically associated with mating. Pheromone signaling is the master regulation system in fungal mating, and it is required for early mating events such as cell recognition, mating projection formation, and initiation of cell contact and cell fusion [47], [48], [49]. However, increasing evidence implies that filamentation in *Cryptococcus* is a plastic process that is not limited to mating or the production of recombinant progeny: Filamentation is occasionally observed under mating-suppressing conditions, even in some attenuated strains isolated from infected host tissues [50], [51], [52], [53]; Filamentation can occur in the absence of some key components of pheromone signaling or meiosis machinery [30], [32], [33], [54]. Thus, filamentation could be used in behaviors unrelated with mating, such as foraging nutrients or defending predation. Such sex-independent cellular differentiation likely involves signaling pathways in response to cues other than the mating signal.

Here we show that sex-independent morphogenesis is linked with virulence in this fungus. We further demonstrate that the transcription factor Znf2 plays a pivotal role in cryptococcal morphological transition, and it is necessary and sufficient to drive filamentation irrespective of environmental cues, mating types, or pheromone signaling. Znf2 not only controls morphogenesis *in vivo*, but also the ability of this fungus to cause diseases. Thus Znf2 provides the key link between morphogenesis and virulence in *Cryptococcus*.

The exact mechanism by which Znf2 controls morphogenesis and links *Cryptococcus* pathogenicity is of great interest. Previous and this current *in vitro* studies indicate that Znf2 does not affect typical *Cryptococcus* virulence traits (e.g. melanization, capsule production, growth at high temperatures, growth in minimal media, and resistance to salt or H_2_O_2_ [42], [55]). Although the P*_GPD1_*-*ZNF2* strain is avirulent, this strain was capable of propagation during the first two weeks of infection (Figure S7). This is in contrast with other avirulent strains such as *cna1* or capsule mutants, which are less fit under various stress conditions and are rapidly cleared by the host [56], [57]. These lines of evidence point to new traits regulated by Znf2 that influence pathogenicity.

Our observation that genes encoding secretory proteins are enriched within the regulon of Znf2 emphasizes the importance of changes in cell surface during morphogenesis. Given that *Cryptococcus* strains with increased flocculation have been noted to be reduced in virulence [40], [41], Znf2 likely impacts pathogenicity at least partly through its effects on cell adhesion (flocculation). Cell adhesion mediated by microbial pathogens usually involves a repertoire of extracellular adhesion proteins. One of Znf2's downstream factors, Cfl1, is a prominent adhesion protein which orchestrates filamentation, cell adhesion, and virulence. To our knowledge, Cfl1 is the first *Cryptococcus* adhesin discovered. Interestingly, Cfl1 does not resemble any known adhesins characterized in ascomycetous fungi in terms of primary sequences and functional domains based on Pfam prediction (http://pfam.sanger.ac.uk/). There are four other homologues of *CFL1* in the genome of *Cryptococcus* and in some other species in the phylum of Basidiomycota (Figure S13), in which no adhesin has been identified so far. This suggests that Cfl1 and its homologues represent a novel adhesion family specific to Basidiomycota.

Unlike Znf2, overexpression of *CFL1* attenuates but does not abolish *Cryptococcus* virulence in the murine model of cryptococcosis. This is not unexpected as studies show that microbes are typically endowed with multiple adhesins. The master regulator Znf2 likely controls additional adhesins and other morphogenesis factors, and it is the orchestrated effects of its downstream targets that give rise to its overall impact on morphogenesis and virulence. Further characterization of Cfl1, other adhesins, and morphogens downstream of Znf2 can help parse out the effects of cell morphotype and other cell properties (e.g. changes in cell surface proteins like adhesins) on *Cryptococcus* virulence. Such investigation may lay a foundation for future endeavors to develop vaccines or alternative therapies against cryptococcosis.

## Materials and Methods

### Ethics Statement

This study was performed according to the guidelines of NIH and Texas A&M University Institutional Animal Care and Use Committee (IACUC). The animal models and procedures used have been approved by the Institutional Animal Care and Use Committee (IACUC) at Texas A&M University (protocol number: 2011-22).

### Strains, Mating, and *in vitro* Phenotypic Assays

Strains used in this study are listed in Table S1. For mating assays, parental strains (**a** and α) with equal number of cells were cocultured together on V8 medium in the dark at 22°C, and mating was examined microscopically for formation of mating hyphae and spores [58]. For cell fusion assays, the coculture of marked parental strains were removed after 48 hours of incubation on V8 medium, washed, and plated on selective media to select fusion products at 37°C as described previously [28], [33], [59]. For self-filamentation assays, cells were patched on V8 medium alone and hypha formation was examined microscopically. Phenotypical assays *in vitro* were performed as previously described [59]. The serotype A strain H99 is highly virulent and has been widely used in pathogenesis studies. Thus strains generated in this genetic background were used in the animal experiments and many of the *in vitro* characterization experiments. However, because wildtype H99 has not been observed to undergo unisexual mating and its bisexual mating is rather weak compared to the well-characterized but less virulent serotype D strains such as JEC21 and XL280, strains generated in these genetic backgrounds were used in some of the morphogenesis and mating assays.

### Construction of Gene Deletion and Gene Overexpression Strains

Plasmids and primers used in this study are listed in Table S2 and S3. For gene deletion, overlap PCR products with an appropriate selection marker connected with the 5′ and 3′ flanking regions of gene of interests were introduced into *Cryptococcus* strains by biolistic transformation and transformants with homologous replacement were selected as described previously [60]. For overexpression, genes were amplified by PCR and the amplified fragments were digested and inserted into pXL1 after the *GPD1* promoter [61]. The P*_GPD1_* of the resulting plasmids was replaced with either the P*_CTR4-2_* or the P*_GAL10_* to generate the copper or the galactose inducible system. The P*_CTR4-2_* and the P*_GAL10_* were amplified from the plasmid pNAT/*CTR4-2* and H99 genomic DNA respectively [35], [62].

### Construction of Fluorescent Proteins and Microscopic Examination

Because Cfl1 contains a predicted secretory signal peptide at its N-terminus, the mCherry [63] was fused to the C-terminus. The fragment including *CFL1* coding region and 1 kb upstream sequences (NCfl1) was pieced together with the mCherry by an overlap PCR. The resulting products were introduced into plasmid pXL1 to generate pXL1-NCfl1-mCherryA (for the serotype A H99 allele) and pXL1-NCfl1-mCherryD (for the serotype D JEC21 allele). The *CFL1-mCherry* without the *CFL1* promoter was amplified and introduced into pXL1 to produce the plasmid pXL1-Cfl1-mCherry. The P*_GPD1_* in pXL1-Cfl1-mCherry was replaced with the P*_CTR4-2_* to generate plasmid pXC-Cfl1-mCherry. To construct overexpression of the fluorescent Cfl1 that lacks the N-terminal signal peptide [Cfl1(sigPΔ)-mCherry], primers primers Linlab948 and Linlab864 were used to generate *CFL1(sigP*Δ*)-mCherry* allele and pXC-Cfl1-mCherry was used as the template. The resulting PCR product was introduced into pXC to produce pXC- Cfl1(sigPΔ)-mCherry. Plasmids were linearized before introduced into relevant *Cryptococcus* strains. To examine the sub-cellular localization of Cfl1::mCherry, strains were grown on V8 agar medium at 22°C for 72 hrs before examined with a BX50 (Olympus) microscope.

### RNA Purification and qPCR Analyses

Total RNA was purified using the purelink RNA purification kit (Invitrogen) and was used as the template for the first strand cDNA synthesis using the Superscript III cDNA synthesis kit (Invitrogen). Relative expression level of selected genes was measured by real time PCR using power SYBR qPCR premix reagents (Invitrogen) in a Realplex system (Eppendorf). Primer efficiency was determined by serially diluting the cDNA and monitoring DNA amplification by real-time PCR. Primers for qPCR used in this study are listed in Table S3. Gene-expression levels were normalized using the endogenous control gene *TEF1*. The relative transcript levels were determined using the comparative CT method as described previously [64].

### Northern Blots

RNA was separated on agarose gels blotted to nylon membrane. Redi-Prime II kit (Amersham) was used to generate probes. The *C. neoformans* actin gene transcript served as a control. mRNA purification was performed using the PolyATtract mRNA Isolation System III (Fisher) according to the manufacture's instruction.

### Measurement of Cryptococcal Biofilms

The cells were cultured in 96-well microtiter plates under a variety of growth conditions. The air-liquid interface biofilm was only observed in *CFL1* overexpression strains. The strains were grown in YPD liquid medium for 8 days. Crystal violet method was used for the quantitative assessment of the ability of *Cryptococcus* strains to form biofilm as previously described [65].

### Murine Models of Cryptococcosis

Animals were infected essentially as previously described [59], [66]. Groups of 6- to 8-week-old female A/J mice (Jackson Labs) were infected intranasally with 1×10^5^ *Cryptococcus* cells in 50 µl PBS. For the P*_GPD1_*-*ZNF2* strain, the culture of cells with mixed morphotype was centrifuged briefly at a low speed to allow the enrichment of yeast cells on the top. The top culture was then centrifuged again and only yeast cells were collected for infection. Ten mice per group were used for survival studies, and four or five were used for organ fungal burden studies and histological examinations. For organ fungal burden studies, fungal CFUs from lungs, kidneys, spleen, and the brains of sacrificed mice at each time point were measured as described previously [59], [67]. Dunnett's two-tailed *t* test was used to test statistical differences (*P*≤0.05). For histological examinations, organs from the sacrificed animals were fixed in 10% formalin, embedded in paraffin, sectioned at 5 µm in thickness, and stained with hematoxylin and eosin (H&E) and Gomori methenamine silver (GMS) as previously described [56], [68]. For mortality studies, the infected animals were monitored until all mice were sacrificed due to sickness or up to DPI 60 when the experiment was terminated. If the experiment was terminated, surviving animals were examined for the presence of *Cryptococcus* cells. Statistical significance (*P*≤0.05) of the survival data between different groups was assessed by the Mantel-Cox log-rank test [69].

### Accession Numbers for Genes and Proteins Mentioned in this Study

*C. neoformans var. grubii* (H99): *ZNF2* (CNAG_03366); *MAT2* (CNAG_06203); *STE3α* (CNAG_06808); *STE6* (CNAG_03600); *MF1α* (CNAG_07407), *CFL1* (CNAG_00795) and other secretory protein encoding genes controlled by Znf2 (CNAG_00596, CNAG_00925, CNAG_01211, CNAG_05778, CNAG_07422, CNAG_06239, CNAG_06411, CNAG_05729), *KEL1* (CNAG_01149), *CDC10* (CNAG_01373), *CDC12* (CNAG_01740), *cnCDC11* (CNAG_02196), *cnMUC1* (CNAG_03234), *cnCDC24* (CNAG_04243), *cnCDC3* (CNAG_05925).

*C. neoformans var. neoformans* (JEC21): *ZNF2* (CNG02160), *MAT2* (CNM02020), STE12α (CND05810), *CFL1* (CNA07720). (Gene ID numbers were obtained from either NCBI Entrez or the *Cryptococcus* genome website at the Broad Institute http://www.broadinstitute.org/annotation/genome/cryptococcus_neoformans/MultiHome.html)

## Supporting Information

Figure S1 **Znf2 governs filamentation in** ***Cryptococcus neoformans*** **.** Wildtype XL280 (serotype D, α) and its derived *znf2*Δ mutant and the P*_GPD1_-ZNF2* strain were grown on V8 juice agar medium at 22°C (mating-inducing condition) (scale bar: 500 µm) or in YPD liquid medium at 30°C (mating-suppressing condition) (scale bar: 25 µm) for 5 days.
(TIF)

Figure S2 **Constitutively activated pheromone signaling is insufficient to maintain hyphal growth under a host-relevant condition.** Wildtype H99 and its derived P*_GPD1_-MAT2* and P*_GPD1_-ZNF2* strains were grown on V8 agar medium at 22°C (mating-inducing condition). At the 5^th^ day, cells were collected, washed, and transferred to serum at 37°C with 5% CO_2_ (host-relevant condition) and incubated for additional 5 days (scale bar: 40 µm). Only cells of the P*_GPD1_-ZNF2* strain remained in the hyphal form under such conditions.
(TIF)

Figure S3 **Znf2 does not control the expression of genes involved in the early events of mating.** Comparative profiling of gene expression in the wildtype, the *mat2*Δ mutant, the *ste7*Δ mutant, and the *znf2*Δ mutant [28] revealed that *S. cerevisiae* homologues known to be involved in early events of mating (e.g. mating projection formation and cell fusion) were regulated by Mat2 and Ste7, but not by Znf2. *CDC3* and *CDC12* were also experimentally shown in *Cryptococcus* to be required for full mating efficiency [63]. The transcript level change (fold) is represented by a color code. The homologues of *C. neoformans* genes in *S. cerevisiae* were identified based on HUWU-BLASTUH program (http://amigo.geneontology.org). “Sce gene” shows the corresponding *S. cerevisiae* gene name of the *C. neoformans* homologue.
(TIF)

Figure S4 **Znf2 controls the level of fungal burden in the brain of infected mice.** Mice were infected intranasally with wildtype H99, the *znf2*Δ mutant, and a P*_GPD1_-ZNF2* strain. Fungal burden in the brains at DPI 10 was determined. Differences among the groups are statistically significant (*p*<0.05). n. d.: Not detectable. CFU: colony forming unit.
(TIF)

Figure S5 **The P** ***_GPD1_-ZNF2*** **strain produces cells of the filamentous form during infection.** Lung tissues from mice infected with *Cryptococcus* strains (H99, the *znf2*Δ mutant and the P*_GPD1_-ZNF2* strain) were fixed, sectioned, and stained with Grocot–Gomori methenamine silver to visualize fungal cells. Scale bar: 10 µm.
(TIF)

Figure S6 **The** ***znf2*** **mutations do not cause any apparent growth defects at high temperature.** (A) Diagram of the P*_CTR4-2_*-*ZNF2* inducible system. (B) Cells of *C. neoformans* strains (H99, *znf2*Δ mutant and P*_CTR4-2_-ZNF2*) were cultured on YPD medium containing CuSO_4_ overnight and all strains were in the yeast form under such condition. The cells then were quantified by measuring the optical density at 600 nm. Three-microliters of the cell suspensions with 10× serial dilutions were spotted onto media. Growth of cells on YPD, DME, and RPMI media containing either BCS or CuSO_4_ at 30°C in the ambient air for 3 days were compared to those at 37°C under 5% CO_2_. Cells grown on DME medium or RPMI medium at 37°C for 3 days appeared more mucoid due to enhanced capsule production. Capsule production was confirmed with India ink staining (data not shown).
(TIF)

Figure S7 **The** ***ZNF2*** **overexpression strain proliferated** ***in vivo*** **.** Mice were infected with the P*_GPD1_-ZNF2* cells in the yeast form intranasally. Fungal burden in the lungs was determined at DPI 1, 7, 12, and 16. The graph shows the changes in fungal burden over time. The average CFU at DPI 1 was 0.66×10^5^.
(TIF)

Figure S8 **The** ***CFL1*** **expression is dependent on Znf2 during bisexual mating.** (A) *CFL1* was highly expressed in **a** x α cocultures under the mating-inducing condition (V8) but not under mating-suppressing conditions (YPD and Serum). H99α and its congenic partner KN99**a** were cocultured on different media for 72 hr. The expression level of *CFL1* during bisexual mating on V8 medium was arbitrarily set as 1 for comparison. (B) The expression pattern of *CFL1* during bilateral matings of the cocultures (**a** X α, **a** z*nf2*Δ X α z*nf2*Δ, **a** *mat2*Δ X α *mat2*Δ and **a** *ste7*Δ X α *ste7*Δ in JEC21 background) on V8 medium (pH = 7.0) for 24 hr.
(TIF)

Figure S9 **Diagram of the m-Cherry labeled wildtype** ***CFL1*** **allele and the mutant** ***CFL1*** **allele that lacks the secretion signal.** Both *CFL1* alleles are constructed under the control of P*_CTR4-2_* so that the transcriptional levels of *CFL1-mCherry* hybrid alleles can be readily manipulated by external addition of inducer (BCS) or inhibitor (CuSO_4_). The arrow points to the 54-bp DNA region predicted to code the secretory signal peptide.
(TIF)

Figure S10 **Overexpression of** ***CFL1*** **results in complex colony morphology and formation of different biofilms.** (A) The *CFL1* overexpression strain and wildtype H99 were grown on YPD agar medium for 4 days. The *CFL1* overexpression strain showed an elaborate pattern of complex multicellular growth. This complex colony morphology resembles the mat biofilm formation reported in *Saccharomyces cerevisiae* [45]. (B and C) The overexpression of *CFL1* greatly enhances the ability of *Cryptococcus* to form different biofilms. *CFL1* overexpression induced by inducer (BCS) triggers the formation of air-liquid interface biofilm (B) and it increases the formation of plastic surface-anchored biofilm (C).
(TIF)

Figure S11 **Overexpression of** ***CFL1*** **results in reduced lung fungal burden.** Mice were infected intranasally with 1×10^5^ cells of either wildtype H99 or the P*_GPD1_-CFL1* strain. Fungal burden in the lungs at DPI 10 was measured. Differences among the groups are statistically significant (*p*<0.05).
(TIF)

Figure S12 **The** ***cfl1*** **mutations do not cause any apparent growth defects at high temperature.** (A) Cells of *C. neoformans* strains (H99, the *cfl1*Δ mutant, and the P*_CTR4-2_-CFL1* strain) were cultured on YPD medium containing CuSO_4_ overnight and all strains were in the yeast form under such condition. The cells then were quantified by determining the optical density at 600 nm. Three-microliters of the cell suspensions with 10× serial dilutions were spotted onto media. Growth of cells on YPD, DME, and RPMI media containing either BCS or CuSO_4_ at 30°C for 3 days in the ambient air were compared to those at 37°C under 5% CO_2_. Notably, cells grown on DME or RPMI medium at 37°C under 5% CO_2_ appeared more mucoid due to enhanced capsule production. Capsule production was confirmed with India ink staining (data not shown). (B) *CFL1* overexpression leads to cell aggregation on RPMI agar.
(TIF)

Figure S13 **Phylogenetic tree of Cfl1 homologs.** Protein sequences were aligned using the neighbor-joining method with MEGA v5.04 program (http://www.megasoftware.net/mega4/mega.html). Cfl1 and its paralogues (Cfl2, Cfl3, Cfl4 and Cfl5) from *Cryptococcus neoformans* are indicated by red dots. Organisms whose genomes contain *CFL1* homologues all belong to the phylum Basidiomycota.
(TIF)

Table S1 **Strains used in this study.**
(DOC)

Table S2 **Plasmids used in this study.**
(DOC)

Table S3 **Primers used in this study.**
(DOC)
