# Supplementary figures and images for: The Link between Morphotype Transition and Virulence in Cryptococcus neoformans
Source: PLoS Pathog. 2012 Jun 21;8(6):e1002765. doi: 10.1371/journal.ppat.1002765 (PMC3380952; doi:10.1371/journal.ppat.1002765)

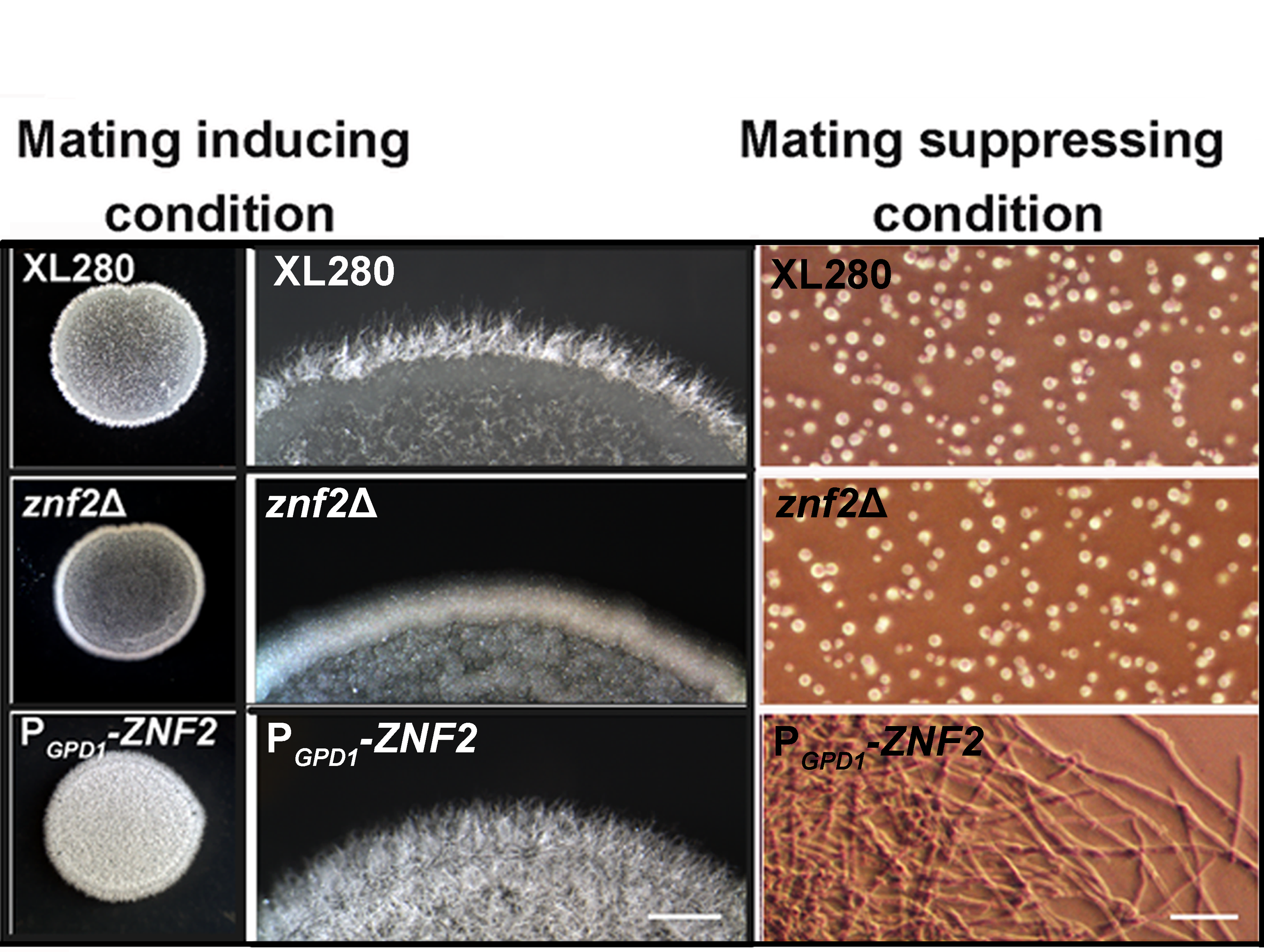

Supplement: Figure S1 — Znf2 governs filamentation in Cryptococcus neoformans . Wildtype XL280 (serotype D, α) and its derived znf2Δ mutant and the PGPD1-ZNF2 strain were grown on V8 juice agar medium at 22°C (mating-inducing condition) (scale bar: 500 µm) or in YPD liquid medium at 30°C (mating-suppressing condition) (scale bar: 25 µm) for 5 days. (TIF) [file ppat.1002765.s001.tif]

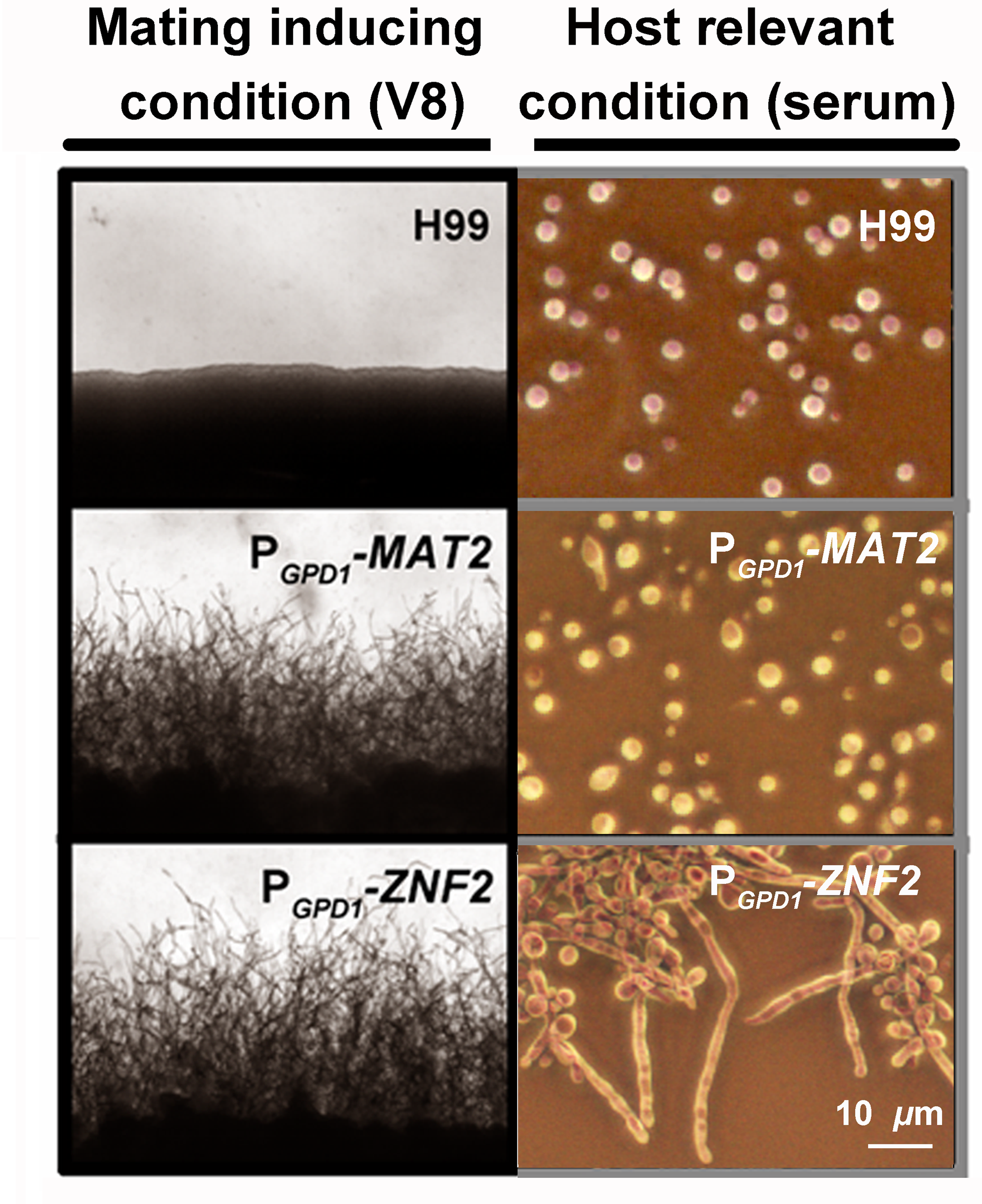

Supplement: Figure S2 — Constitutively activated pheromone signaling is insufficient to maintain hyphal growth under a host-relevant condition. Wildtype H99 and its derived PGPD1-MAT2 and PGPD1-ZNF2 strains were grown on V8 agar medium at 22°C (mating-inducing condition). At the 5th day, cells were collected, washed, and transferred to serum at 37°C with 5% CO2 (host-relevant condition) and incubated for additional 5 days (scale bar: 40 µm). Only cells of the PGPD1-ZNF2 strain remained in the hyphal form under such conditions. (TIF) [file ppat.1002765.s002.tif]

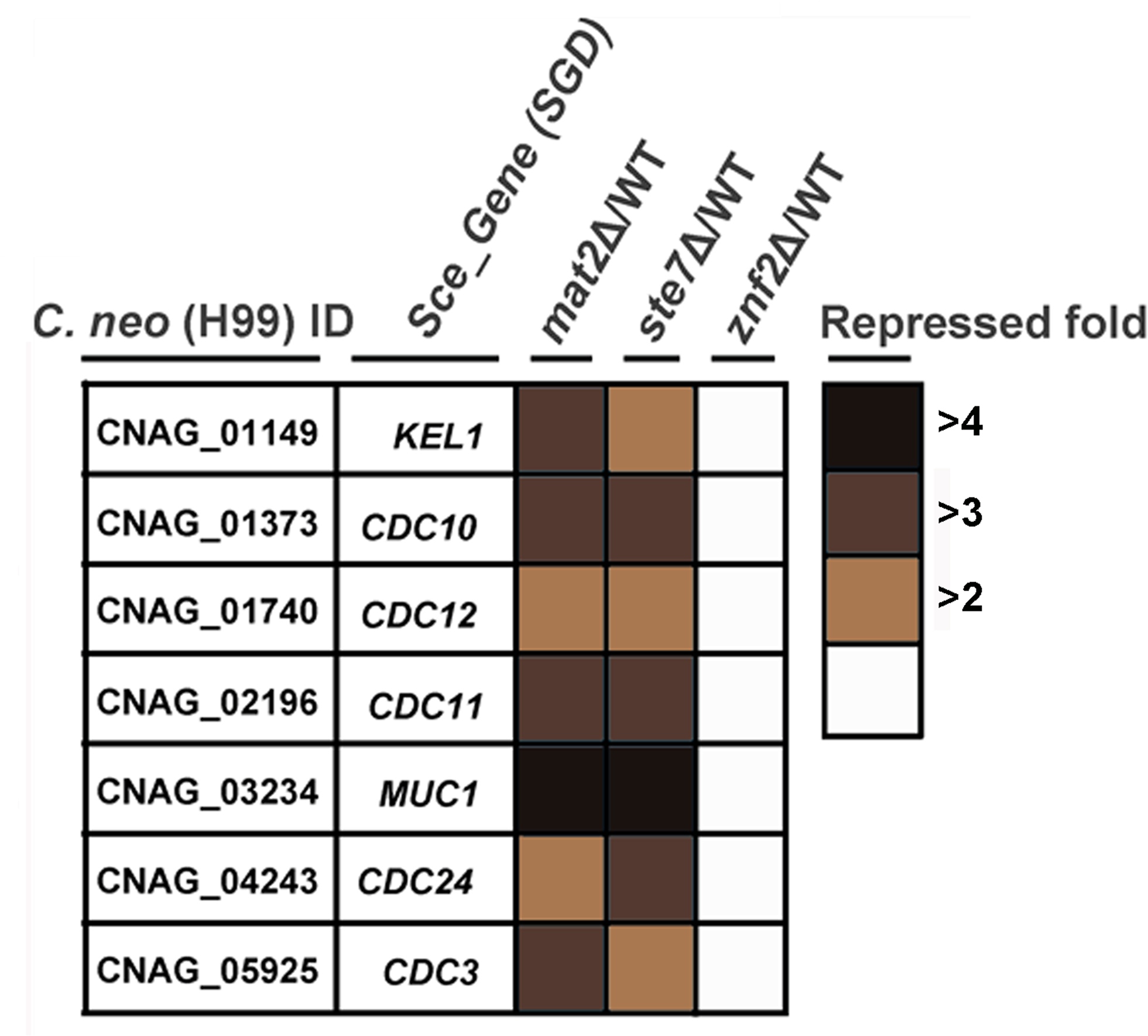

Supplement: Figure S3 — Znf2 does not control the expression of genes involved in the early events of mating. Comparative profiling of gene expression in the wildtype, the mat2Δ mutant, the ste7Δ mutant, and the znf2Δ mutant [28] revealed that S. cerevisiae homologues known to be involved in early events of mating (e.g. mating projection formation and cell fusion) were regulated by Mat2 and Ste7, but not by Znf2. CDC3 and CDC12 were also experimentally shown in Cryptococcus to be required for full mating efficiency [63]. The transcript level change (fold) is represented by a color code. The homologues of C. neoformans genes in S. cerevisiae were identified based on HUWU-BLASTUH program (http://amigo.geneontology.org). “Sce gene” shows the corresponding S. cerevisiae gene name of the C. neoformans homologue. (TIF) [file ppat.1002765.s003.tif]

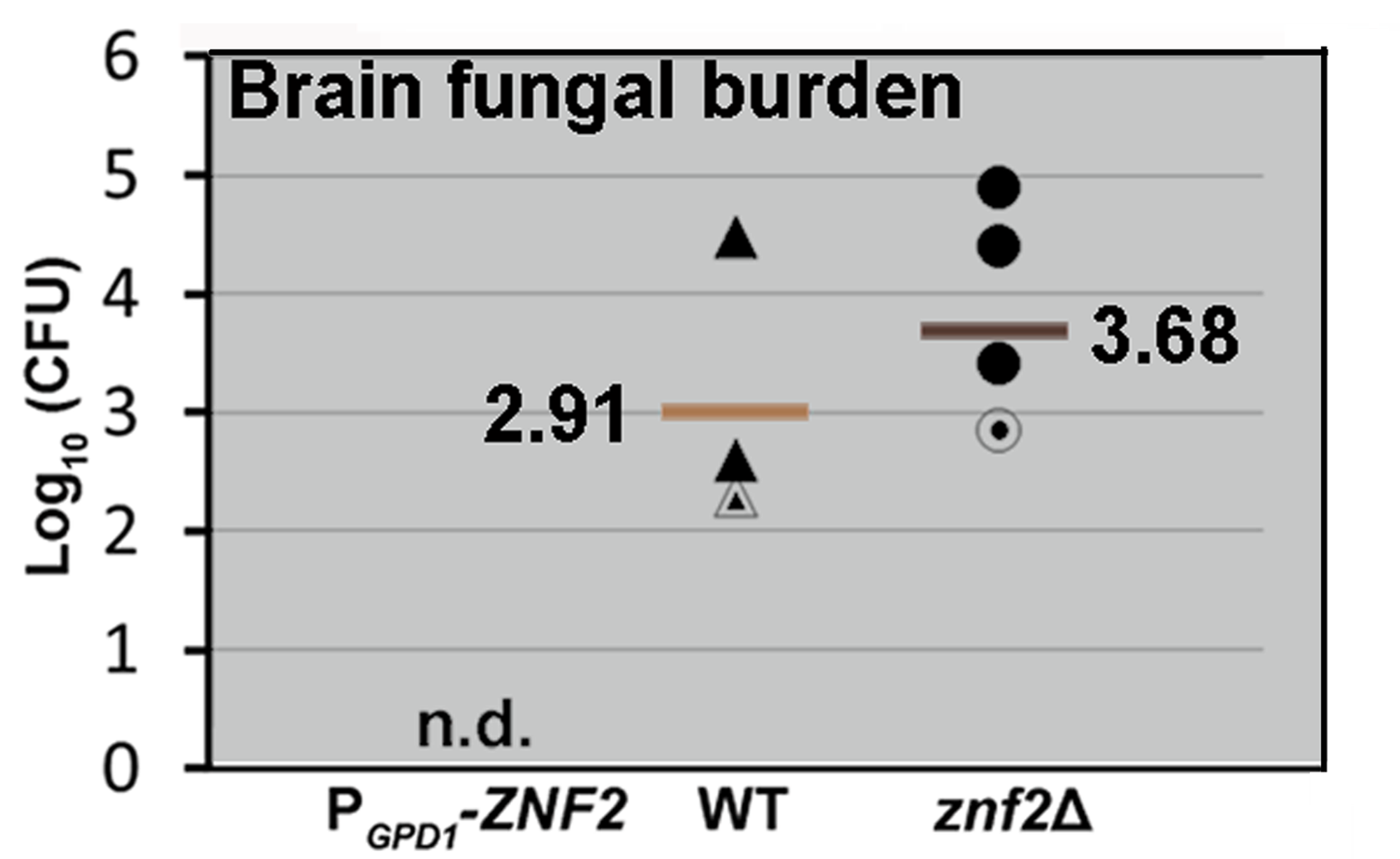

Supplement: Figure S4 — Znf2 controls the level of fungal burden in the brain of infected mice. Mice were infected intranasally with wildtype H99, the znf2Δ mutant, and a PGPD1-ZNF2 strain. Fungal burden in the brains at DPI 10 was determined. Differences among the groups are statistically significant (p<0.05). n. d.: Not detectable. CFU: colony forming unit. (TIF) [file ppat.1002765.s004.tif]

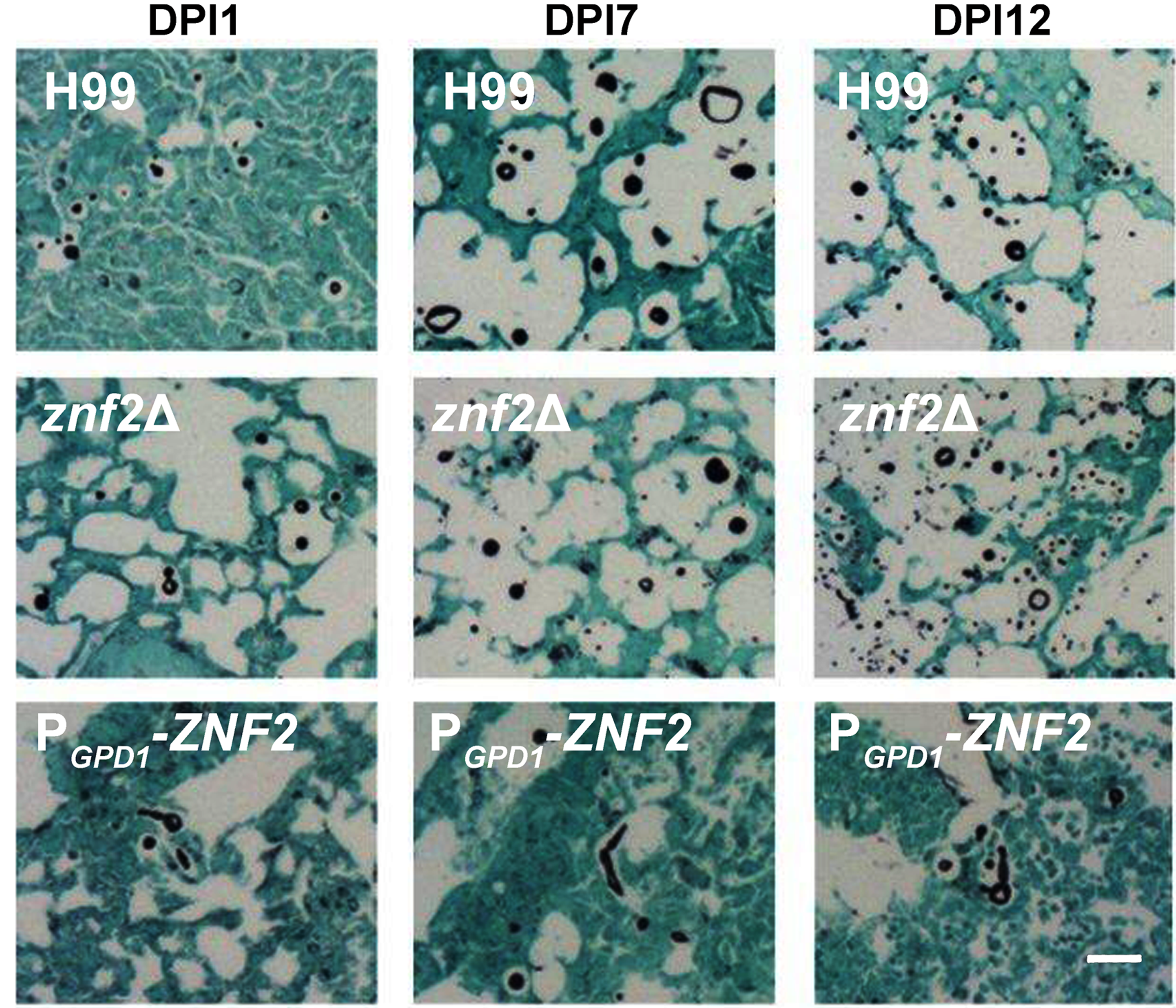

Supplement: Figure S5 — The P GPD1-ZNF2 strain produces cells of the filamentous form during infection. Lung tissues from mice infected with Cryptococcus strains (H99, the znf2Δ mutant and the PGPD1-ZNF2 strain) were fixed, sectioned, and stained with Grocot–Gomori methenamine silver to visualize fungal cells. Scale bar: 10 µm. (TIF) [file ppat.1002765.s005.tif]

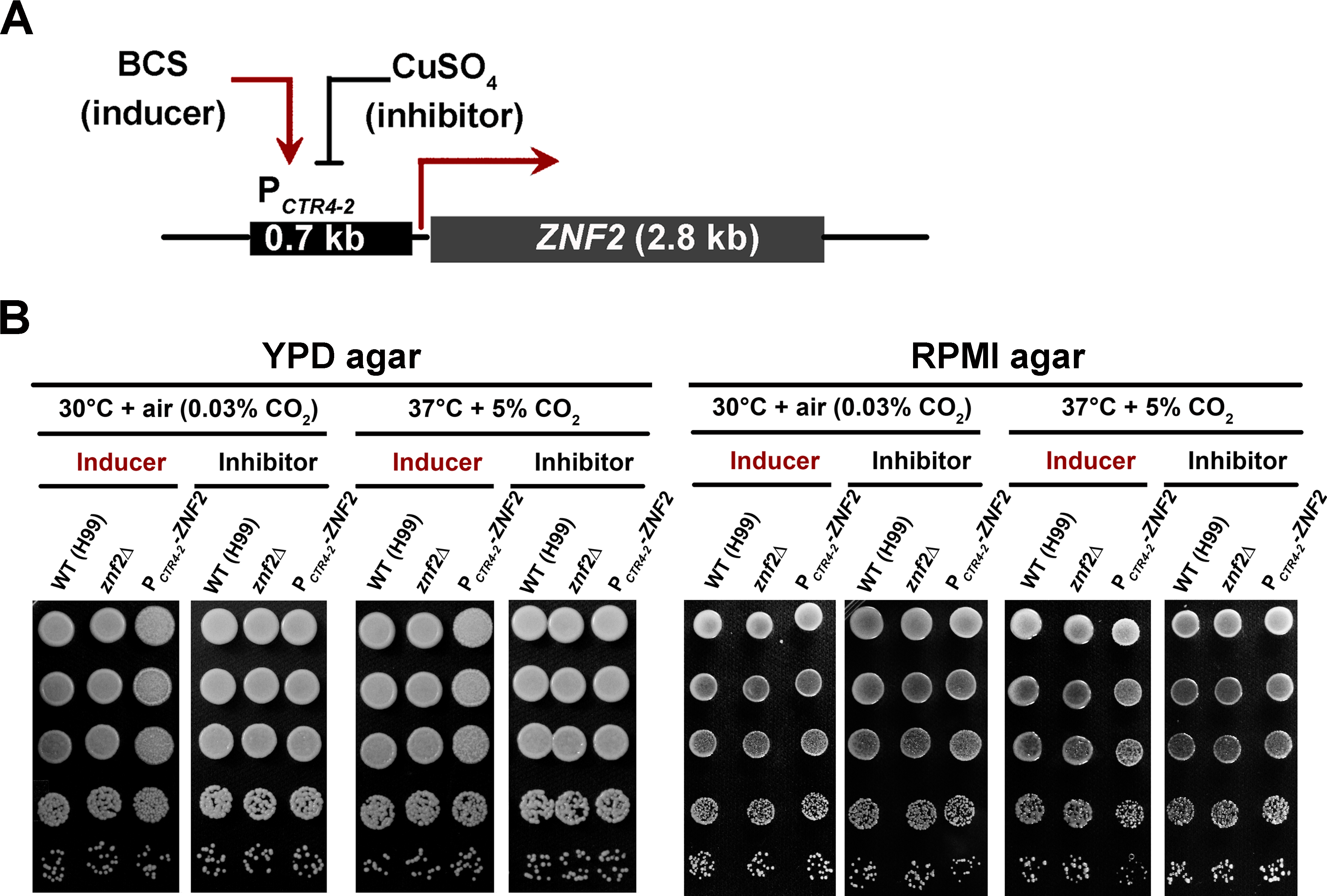

Supplement: Figure S6 — The znf2 mutations do not cause any apparent growth defects at high temperature. (A) Diagram of the PCTR4-2-ZNF2 inducible system. (B) Cells of C. neoformans strains (H99, znf2Δ mutant and PCTR4-2-ZNF2) were cultured on YPD medium containing CuSO4 overnight and all strains were in the yeast form under such condition. The cells then were quantified by measuring the optical density at 600 nm. Three-microliters of the cell suspensions with 10× serial dilutions were spotted onto media. Growth of cells on YPD, DME, and RPMI media containing either BCS or CuSO4 at 30°C in the ambient air for 3 days were compared to those at 37°C under 5% CO2. Cells grown on DME medium or RPMI medium at 37°C for 3 days appeared more mucoid due to enhanced capsule production. Capsule production was confirmed with India ink staining (data not shown). (TIF) [file ppat.1002765.s006.tif]

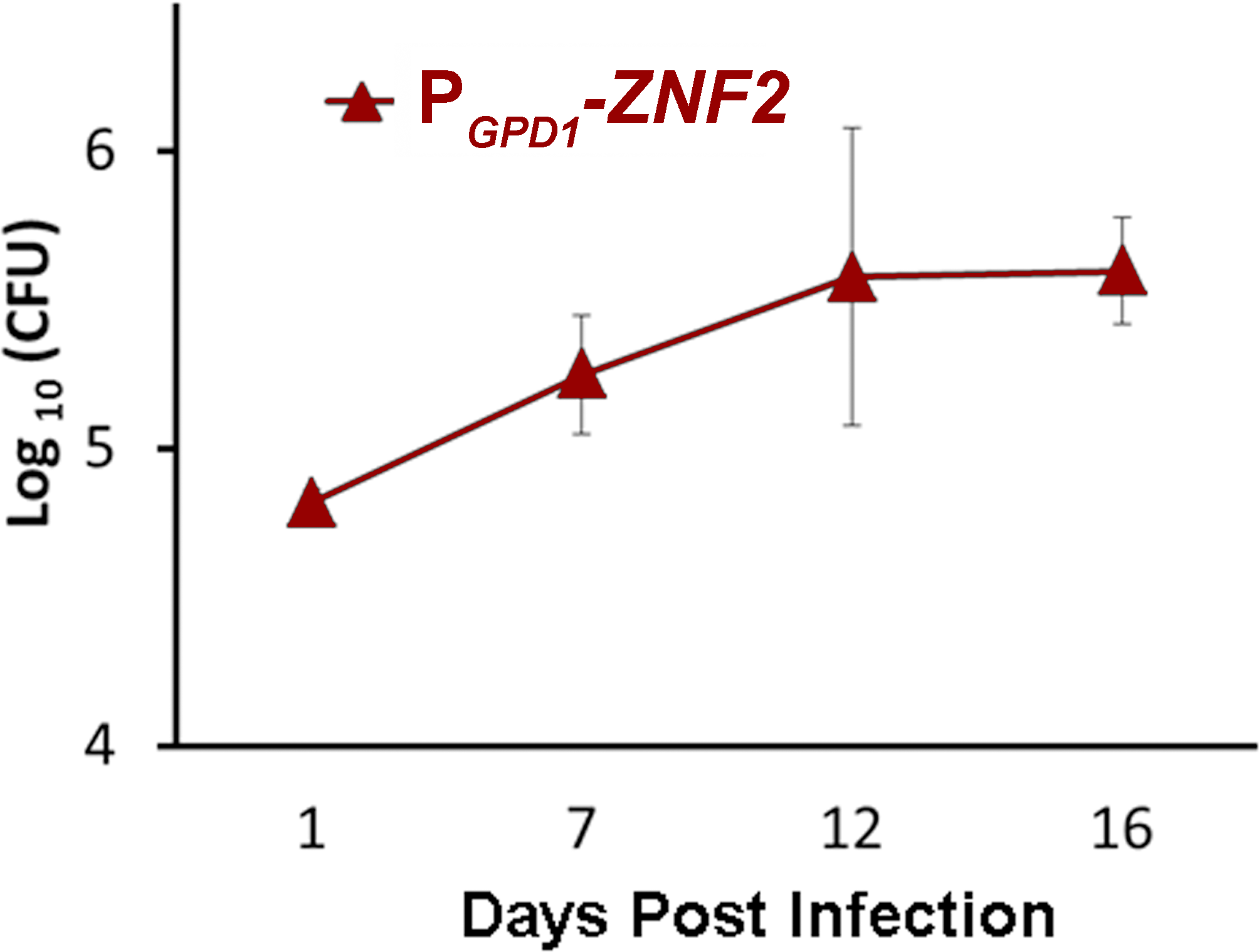

Supplement: Figure S7 — The ZNF2 overexpression strain proliferated in vivo . Mice were infected with the PGPD1-ZNF2 cells in the yeast form intranasally. Fungal burden in the lungs was determined at DPI 1, 7, 12, and 16. The graph shows the changes in fungal burden over time. The average CFU at DPI 1 was 0.66×10^5^. (TIF) [file ppat.1002765.s007.tif]

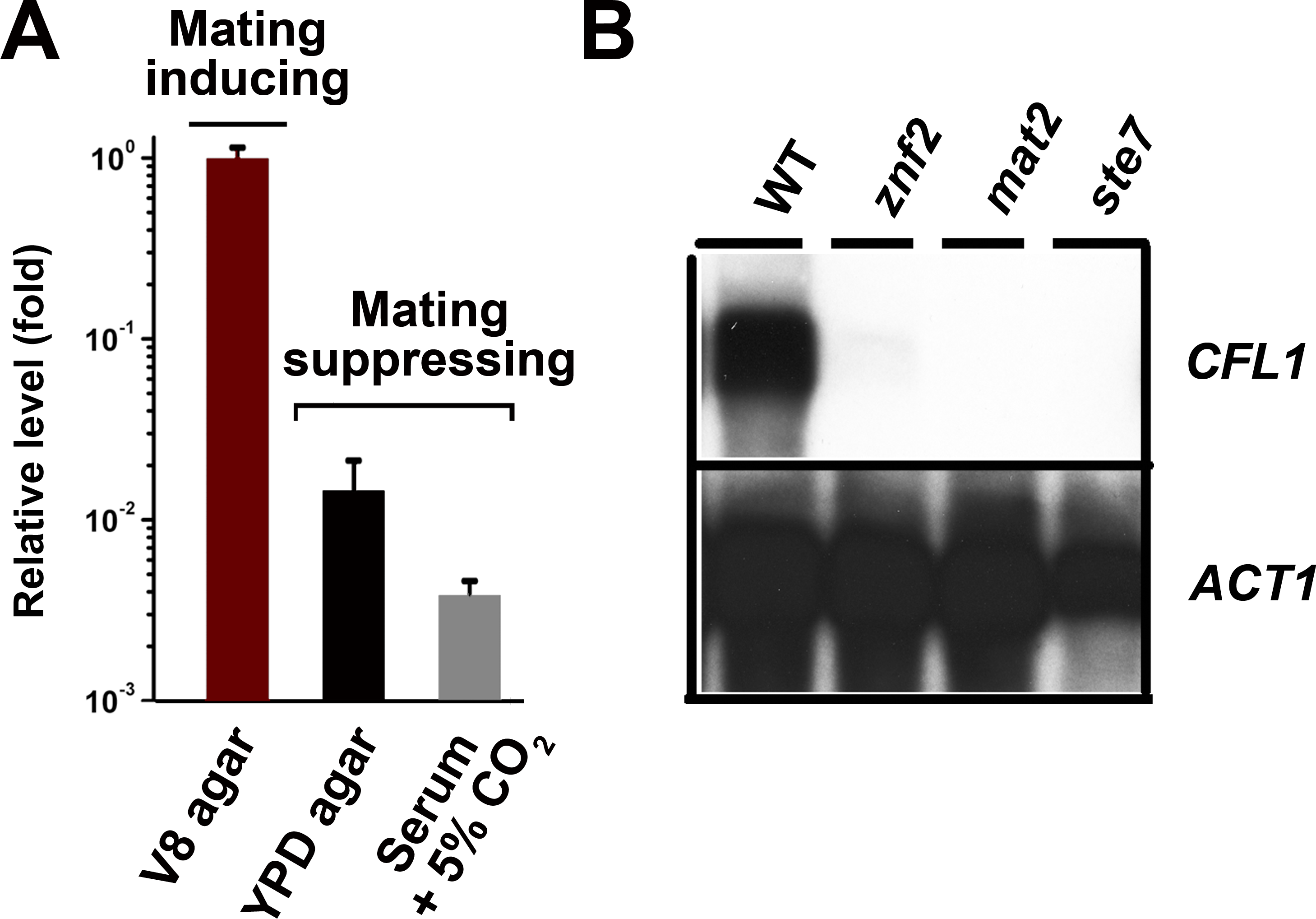

Supplement: Figure S8 — The CFL1 expression is dependent on Znf2 during bisexual mating. (A) CFL1 was highly expressed in a x α cocultures under the mating-inducing condition (V8) but not under mating-suppressing conditions (YPD and Serum). H99α and its congenic partner KN99a were cocultured on different media for 72 hr. The expression level of CFL1 during bisexual mating on V8 medium was arbitrarily set as 1 for comparison. (B) The expression pattern of CFL1 during bilateral matings of the cocultures (a X α, a znf2Δ X α znf2Δ, a mat2Δ X α mat2Δ and a ste7Δ X α ste7Δ in JEC21 background) on V8 medium (pH = 7.0) for 24 hr. (TIF) [file ppat.1002765.s008.tif]

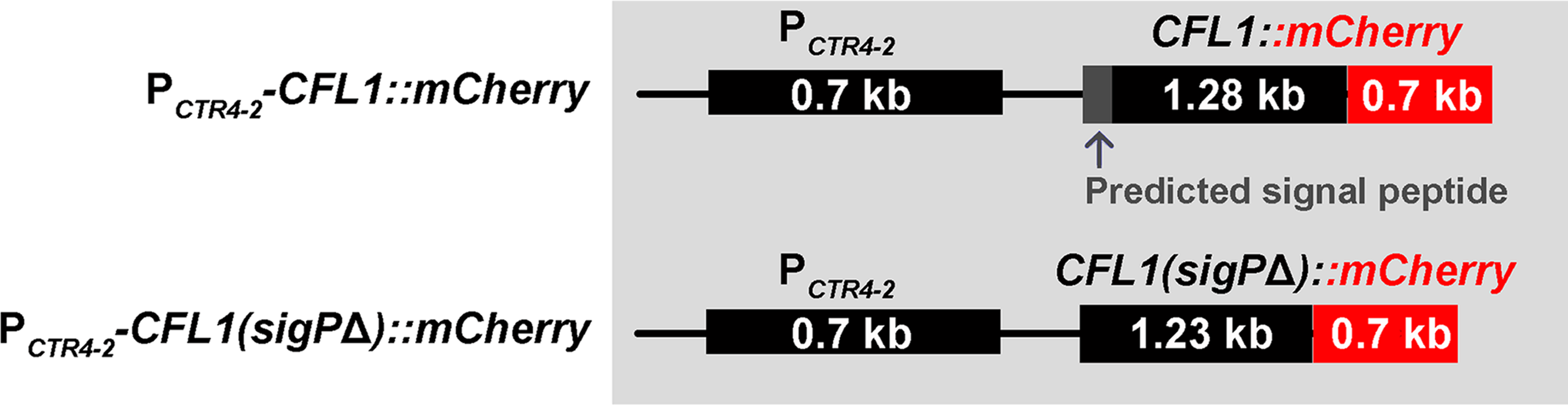

Supplement: Figure S9 — Diagram of the m-Cherry labeled wildtype CFL1 allele and the mutant CFL1 allele that lacks the secretion signal. Both CFL1 alleles are constructed under the control of PCTR4-2 so that the transcriptional levels of CFL1-mCherry hybrid alleles can be readily manipulated by external addition of inducer (BCS) or inhibitor (CuSO4). The arrow points to the 54-bp DNA region predicted to code the secretory signal peptide. (TIF) [file ppat.1002765.s009.tif]

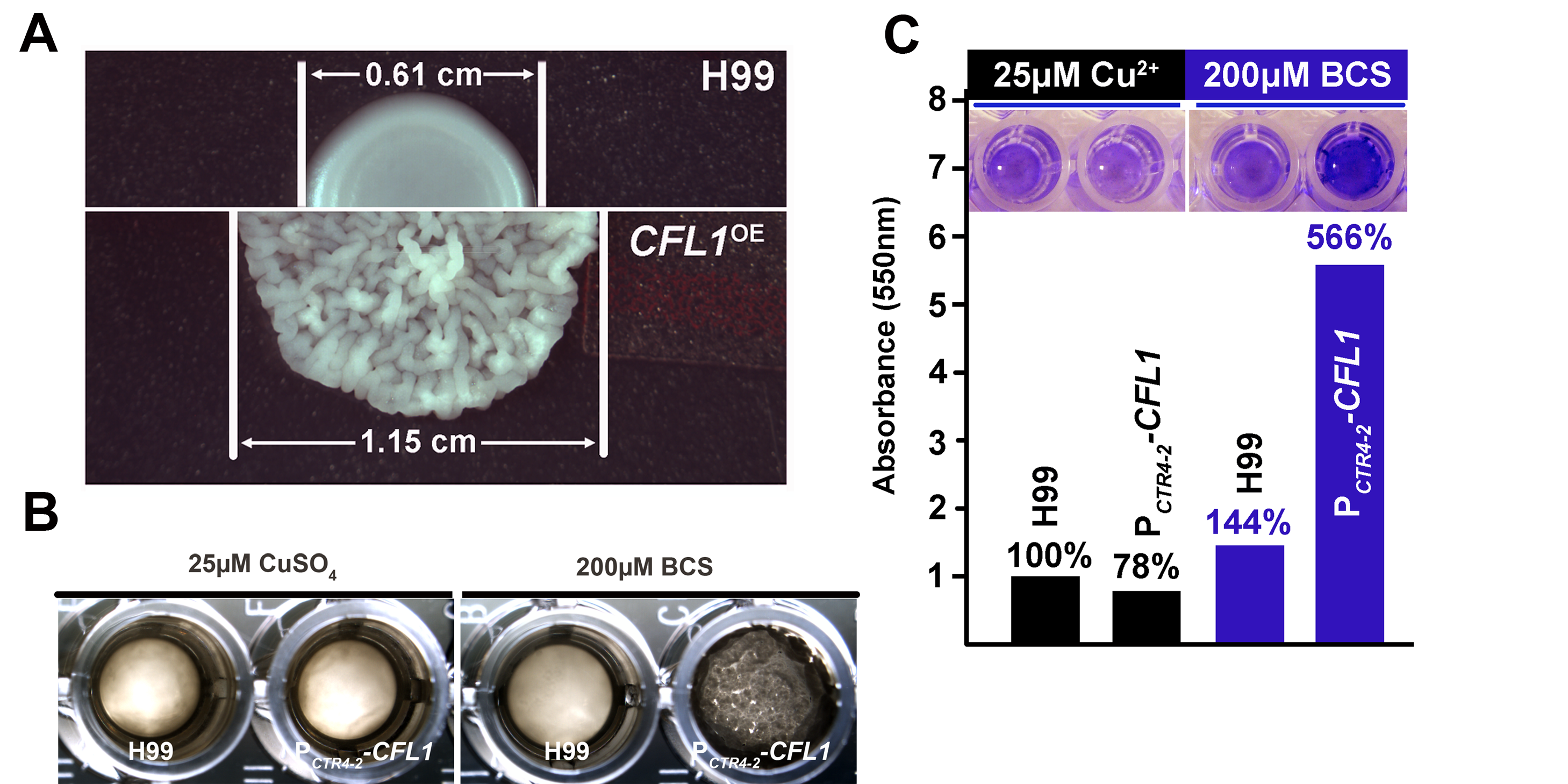

Supplement: Figure S10 — Overexpression of CFL1 results in complex colony morphology and formation of different biofilms. (A) The CFL1 overexpression strain and wildtype H99 were grown on YPD agar medium for 4 days. The CFL1 overexpression strain showed an elaborate pattern of complex multicellular growth. This complex colony morphology resembles the mat biofilm formation reported in Saccharomyces cerevisiae [45]. (B and C) The overexpression of CFL1 greatly enhances the ability of Cryptococcus to form different biofilms. CFL1 overexpression induced by inducer (BCS) triggers the formation of air-liquid interface biofilm (B) and it increases the formation of plastic surface-anchored biofilm (C). (TIF) [file ppat.1002765.s010.tif]

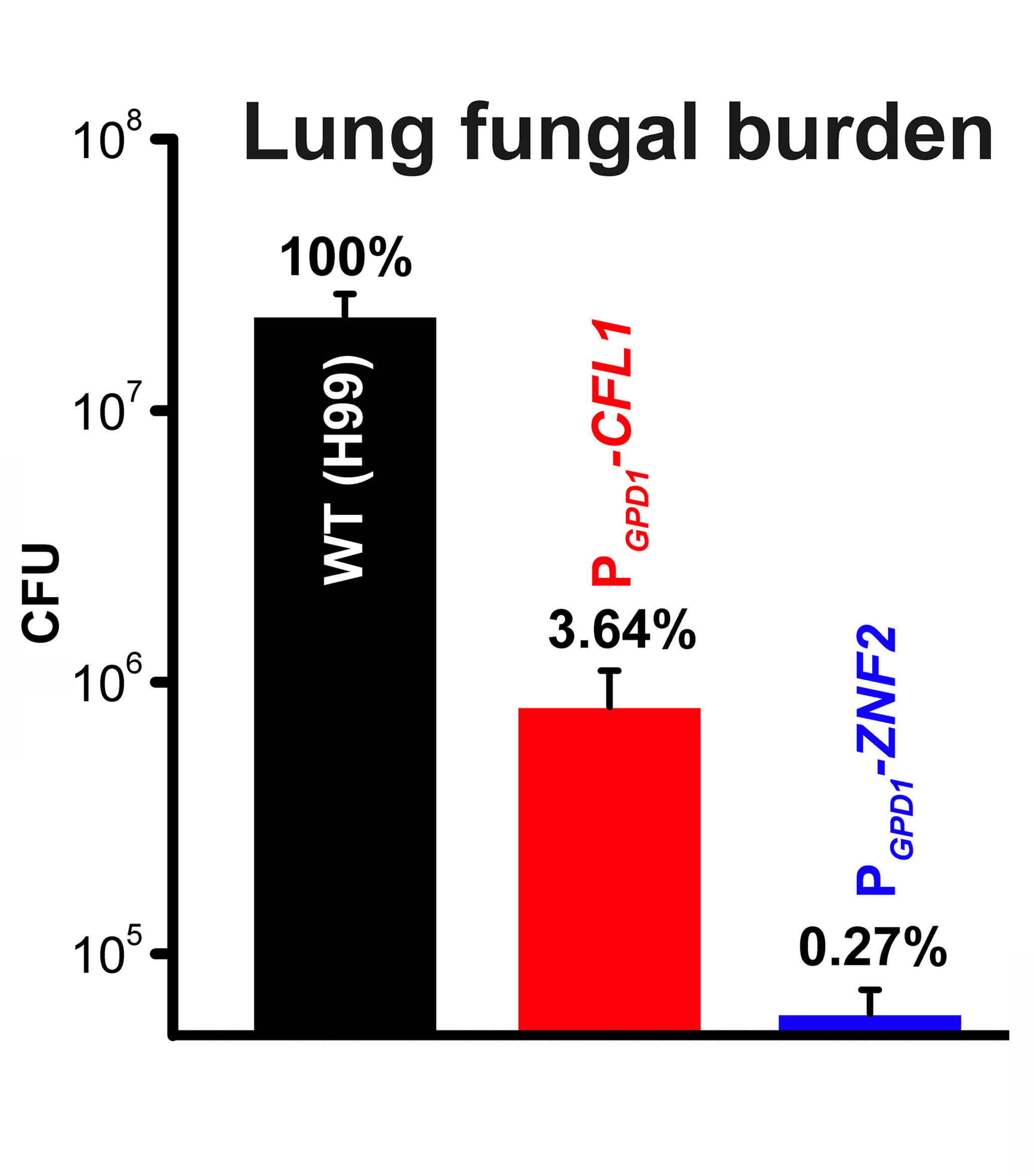

Supplement: Figure S11 — Overexpression of CFL1 results in reduced lung fungal burden. Mice were infected intranasally with 1×10^5^ cells of either wildtype H99 or the PGPD1-CFL1 strain. Fungal burden in the lungs at DPI 10 was measured. Differences among the groups are statistically significant (p<0.05). (TIF) [file ppat.1002765.s011.tif]

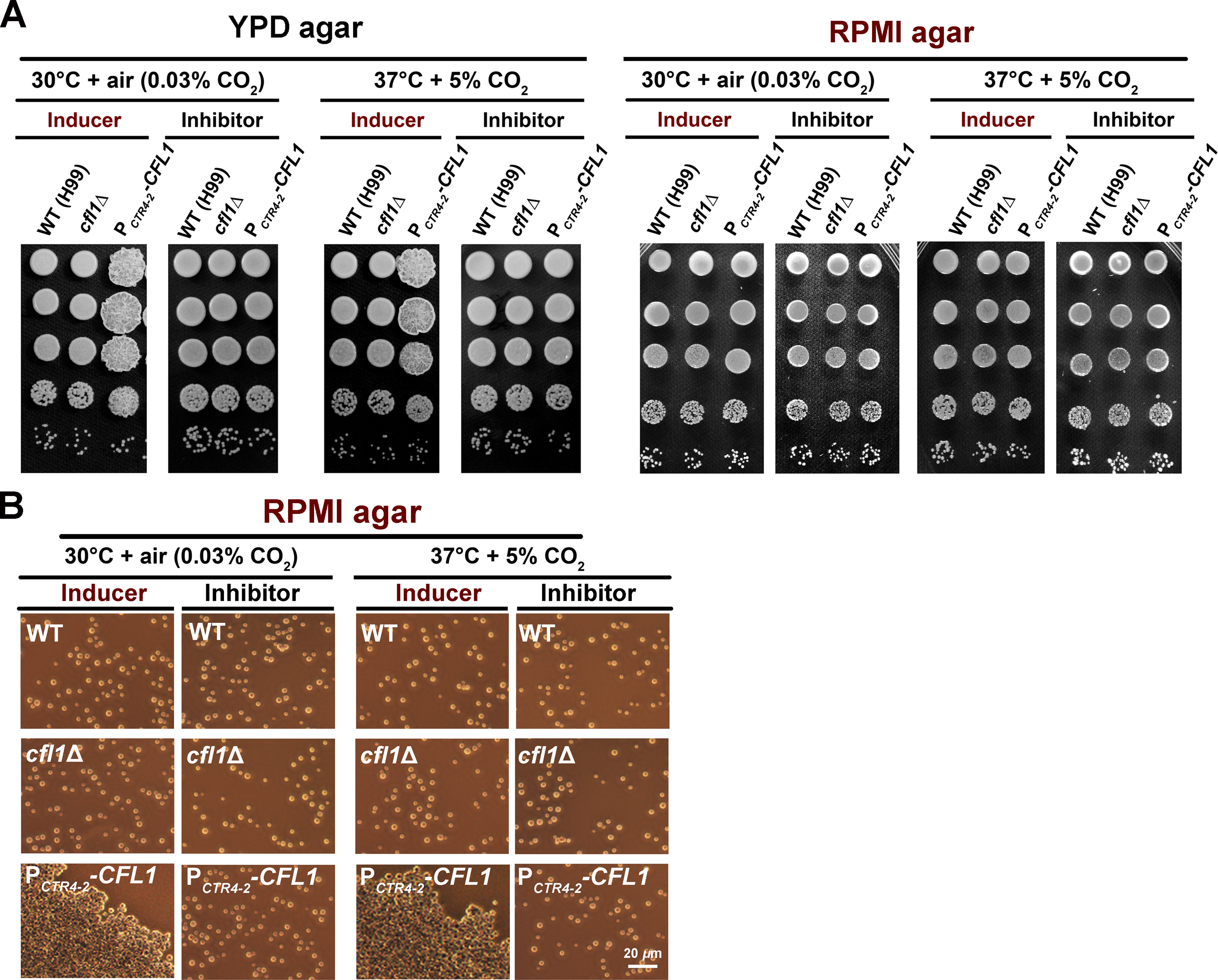

Supplement: Figure S12 — The cfl1 mutations do not cause any apparent growth defects at high temperature. (A) Cells of C. neoformans strains (H99, the cfl1Δ mutant, and the PCTR4-2-CFL1 strain) were cultured on YPD medium containing CuSO4 overnight and all strains were in the yeast form under such condition. The cells then were quantified by determining the optical density at 600 nm. Three-microliters of the cell suspensions with 10× serial dilutions were spotted onto media. Growth of cells on YPD, DME, and RPMI media containing either BCS or CuSO4 at 30°C for 3 days in the ambient air were compared to those at 37°C under 5% CO2. Notably, cells grown on DME or RPMI medium at 37°C under 5% CO2 appeared more mucoid due to enhanced capsule production. Capsule production was confirmed with India ink staining (data not shown). (B) CFL1 overexpression leads to cell aggregation on RPMI agar. (TIF) [file ppat.1002765.s012.tif]

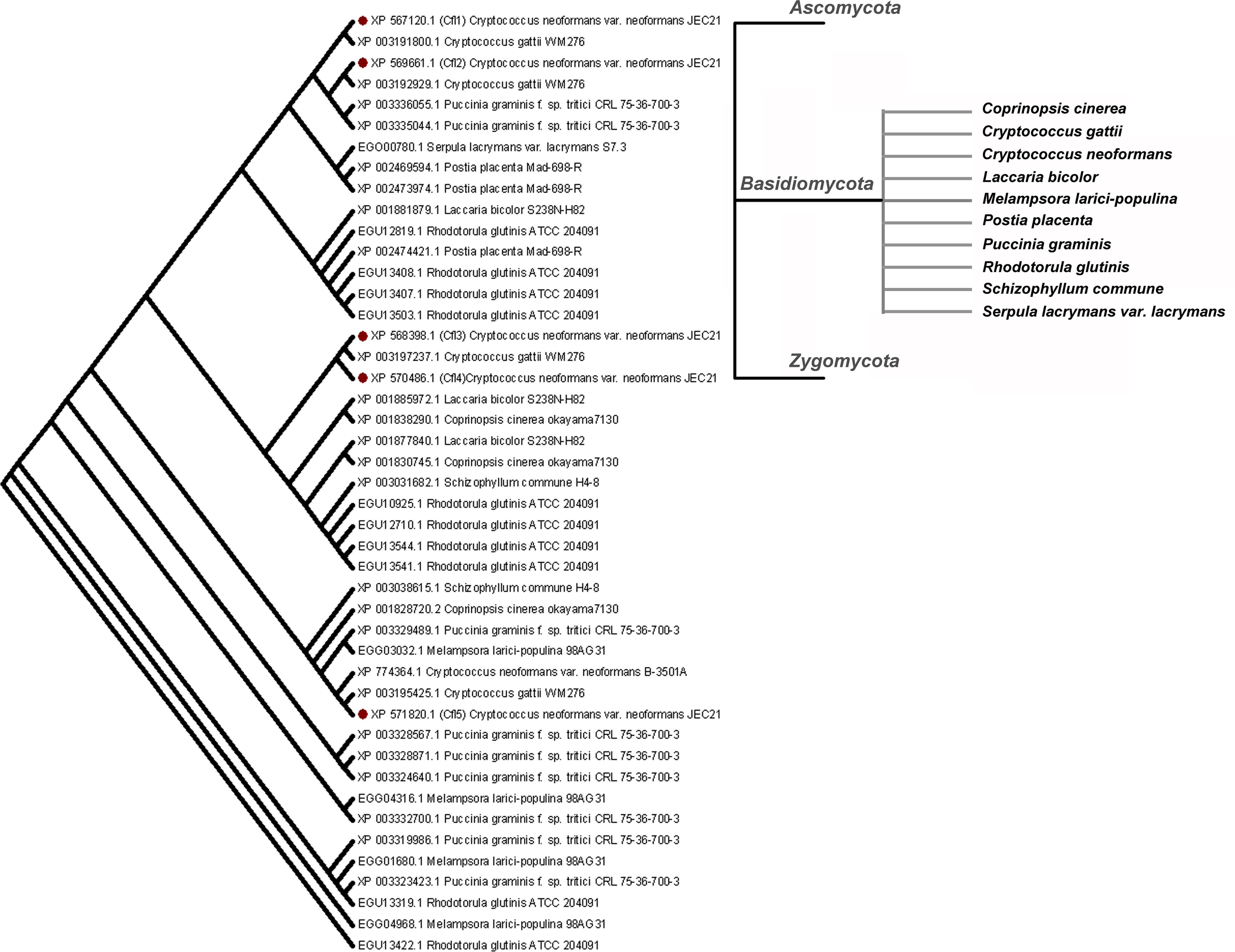

Supplement: Figure S13 — Phylogenetic tree of Cfl1 homologs. Protein sequences were aligned using the neighbor-joining method with MEGA v5.04 program (http://www.megasoftware.net/mega4/mega.html). Cfl1 and its paralogues (Cfl2, Cfl3, Cfl4 and Cfl5) from Cryptococcus neoformans are indicated by red dots. Organisms whose genomes contain CFL1 homologues all belong to the phylum Basidiomycota. (TIF) [file ppat.1002765.s013.tif]
