# Supplementary material for: The Link between Morphotype Transition and Virulence in Cryptococcus neoformans
Source: PLoS Pathog. 2012 Jun 21;8(6):e1002765. doi: 10.1371/journal.ppat.1002765 (PMC3380952; doi:10.1371/journal.ppat.1002765)
Supplement: Table S1 — Strains used in this study. (DOC) [file ppat.1002765.s014.doc]

**Table S1. Strains used in this study.**

| **Strain name** | **Genotype** | **Source and comments** |
| --- | --- | --- |
| JEC21α | wild type | [1,2] |
| JEC20**a** | wild type | Congenic with JEC21 [1,2] |
| JEC169**a** ^1^ | *ade2 lys1 ura5* | [3] |
| XL330α ^1^ | *ura5* | This study |
| XL878**a** ^1^ | *lys1* | [4] |
| WSC18α ^1^ | *mfα1::ADE2 mfα23::URA5* | [5] |
| XL975α ^1^ | *mat2::NAT, ura5* | [4] |
| JH# 926α ^1^ | *ste12α::URA5 ura5* | From J Heitman |
| LW23α ^1^ | *Δmfα1::ADE2 Δmfα23::URA5,* P*_GPD1_-ZNF2:: NEO^r^* | WSC18α transformed with pXL1-ZNF2 that bears the *ZNF2* gene with the *GPD1* promoter [6]. |
| LW37α ^1^ | *mat2:: NAT^r^,* P*_GPD1_-ZNF2:: NEO^r^ ura5* | XL975α transformed with pXL1-ZNF2 that bears the *ZNF2* gene with the *GPD1* promoter [6]. |
| LW38α ^1^ | *ste12::URA5,* P*_GPD1_-ZNF2:: NEO^r^* | JH# 926α transformed with pXL1-ZNF2 that bears the *ZNF2* gene with the *GPD1* promoter. |
| LW181α ^1^ | P*_CFL1_-CFL1::mCherry:: NEO^r^* | This study |
| LW188**a** ^1^ | P*_CFL1_-CFL1::mCherry:: NEO^r^* | This study |
| XL1448 **a** ^1^ | *Cfl1*::*NEO^r^* | This study |
| XL1449α ^1^ | *Cfl1*::*NEO^r^* | This study |
| LW110α ^1^ | *znf2*::*NAT^r^* P*_GPD1_-MAT2:: NEO^r^* | XL576α [4] transformed with pXL1-MAT2 that bears the *MAT2* gene with the *GPD1* promoter [6]. |
| LW103α ^1^ | *znf2*::*NAT^r^* P*_GPD1_-STE12:: NEO^r^* | XL576α [4] transformed with pXL1-STE12 that bears the *STE12* gene with the *GPD1* promoter [6]. |
| XL576α ^1^ | *znf2*::*NAT^r^* | [4] |
| XL926α ^1^ | *mat2::NAT^r^* | [4] |
| XL975α ^1^ | *mat2::NAT^r^ ura5* | [4] |
| H99 | wild type | [7] |
| KN99α ^2^ | wild type | Isogenic with H99 [8] |
| KN99**a** ^2^ | wild type | Congenic with H99/KN99α [8] |
| LW10α ^2^ | P*_GPD1_-ZNF2:: NEO^r^* | This study |
| LW167**a** ^2^ | P*_GPD1_-ZNF2:: NEO^r^* | This study |
| LW28α ^2^ | P*_CTR4-2-_ZNF2:: NEO^r^* | This study |
| LW39α ^2^ | P*_GAL10_-ZNF2:: NEO^r^* | This study |
| LW80α ^2^ | P*_GPD1_-MAT2:: NEO^r^* | This study |
| LW87α ^2^ | P*_GPD1_-CFL1:: NEO^r^* | This study |
| LW82α ^2^ | P*_CTR4-2_-CFL1:: NEO^r^* | This study |
| LW130α ^2^ | P*_CTR4-2-_*CNAG-00925*:: NEO^r^* | This study |
| LW48α ^2^ | P*_GPD1-_* CNAG-00596*:: NEO^r^* | This study |
| LW169α ^2^ | P*_CTR4-2-_* CNAG-05778*:: NEO^r^* | This study |
| LW73α ^2^ | P*_GPD1-_* CNAG-07422*:: NEO^r^* | This study |
| LW177α ^2^ | P*_CTR4-2-_* CNAG-06239*:: NEO^r^* | This study |
| LW67α ^2^ | P*_GPD1-_* CNAG-06411*:: NEO^r^* | This study |
| LW66α ^2^ | P*_GPD1-_* CNAG-05729*:: NEO^r^* | This study |
| LW173α ^2^ | P*_CTR4-2-_* CNAG-01121*:: NEO^r^* | This study |
| LW204α ^2^ | P*_CTR4-2_-CFL1::mCherry:: NEO^r^* | This study |
| XL1601α ^2^ | *znf2*::*NEO^r^* | [4] |
| XL280α ^3^ | wild type | [9] |
| XL1359α ^3^ | *cfl1*::*NAT^r^* | This study |
| XL574α ^3^ | *znf2*::*NAT^r^* | [4] |
| LW1α ^3^ | *znf2*::*NAT^r^* P*_GPD1_-ZNF2:: NEO^r^* | XL574α [4] transformed with pXL1-ZNF2D that bears the *ZNF2* gene with the *GPD1* promoter [6]. |
| LW31α ^3^ | *znf2*::*NAT^r^* P*_CTR4-2_-ZNF2:: NEO^r^* | XL574α [4] transformed with pXC1-ZNF2D that bears the *ZNF2* gene with the *CTR4-2* promoter [10]. |
| LW42α ^3^ | P*_GAL10_-ZNF2:: NEO^r^* | This study |
| LW206α ^3^ | P*_GPD1_-CFL1:: NEO^r^* | This study |
| LW192α ^3^ | P*_CFL1_-CFL1::mCherry:: NEO^r^* | This study |
| LW250α ^3^ | *znf2::NAT^r^,* P*_CFL1_-CFL1::mCherry:: HYG^r^,* P*_GAL10_-ZNF2:: NEO^r^* | This study |
| LW260α ^3^ | P*_CFL1_-CFL1(sigP*Δ*)::mCherry:: NEO^r^* | This study |
| XL574α ^3^ | *znf2*::*NAT^r^* | [4] |

^1^: Strains in JEC21/JEC20 background (serotype D)

^2^: Strains in H99α/KN99**a** background (serotype A)

^3^: Strains in XL280 background (serotype D)

1. Kwon-Chung KJ, Kozel TR, Edman JC, Polacheck I, Ellis D, et al. (1992) Recent

advances in biology and immunology of *Cryptococcus neoformans*. J Med Vet Mycol 30 Suppl 1: 133-142.

2. Heitman J, Allen B, Alspaugh JA, Kwon-Chung KJ (1999) On the origins of

congenic *MAT*a and *MAT***a** strains of the pathogenic yeast *Cryptococcus*

*neoformans*. Fungal Genet Biol 28: 1-5.

3. Moore TD, Edman JC (1993) The a-mating type locus of *Cryptococcus neoformans*

contains a peptide pheromone gene. Mol Cell Biol 13: 1962-1970.

4. Lin X, Jackson JC, Feretzaki M, Xue C, Heitman J (2010) Transcription factors

Mat2 and Znf2 operate cellular circuits orchestrating opposite and same-sex mating

in *Cryptococcus neoformans*. PLoS Genet 6: e1000953.

5. Shen WC, Davidson RC, Cox GM, Heitman J (2002) Pheromones stimulate mating

and differentiation *via* paracrine and autocrine signaling in *Cryptococcus*

*neoformans*. Eukaryot Cell 1: 366-377.

6. Hsueh YP, Xue C, Heitman J (2009) A constitutively active GPCR governs

morphogenic transitions in *Cryptococcus neoformans*. EMBO J 28: 1220-1233.

7. Perfect JR, Lang SD, Durack DT (1980) Chronic cryptococcal meningitis: a new

experimental model in rabbits. Am J Pathol 101: 177-194.

8. Nielsen K, Cox GM, Wang P, Toffaletti DL, Perfect JR, et al. (2003) Sexual cycle

of *Cryptococcus neoformans var. grubii* and virulence of congenic **a** and a isolates.

Infect Immun 71: 4831-4841.

9. Lin X, Huang JC, Mitchell TG, Heitman J (2006) Virulence attributes and hyphal

growth of *C. neoformans* are quantitative traits and the *MAT*a allele enhances

filamentation. PLoS Genet 2: e187.

10. Ory JJ, Griffith CL, Doering TL (2004) An efficiently regulated promoter system

for *Cryptococcus neoformans* utilizing the *CTR4* promoter. Yeast 21: 919-926.
