# Supplementary material for: The Link between Morphotype Transition and Virulence in Cryptococcus neoformans
Source: PLoS Pathog. 2012 Jun 21;8(6):e1002765. doi: 10.1371/journal.ppat.1002765 (PMC3380952; doi:10.1371/journal.ppat.1002765)
Supplement: Table S2 — Plasmids used in this study. (DOC) [file ppat.1002765.s015.doc]

**Table S2. Plasmids used in this study.**

| **Strain name** |  | **Source and comments** |
| --- | --- | --- |
| pXL1 | P*_GPD1_* *NEO^r^ Kan^R^* | [1] |
| pNAT/CTR4-2 | P*_CTR4-2_* *NEO^r^ Amp^R^* | [2] |
| pXL1-ZNF2A | P*_GPD1-_ZNF2* (H99) *NEO^r^ Kan^R^* | This study |
| pXL1-ZNF2D | P*_GPD1-_ZNF2* (JEC21) *NEO^r^ Kan^R^* | This study |
| pXC-ZNF2A | P*_CTR4-2-_ZNF2* (H99) *NEO^r^ Kan^R^* | This study |
| pXC-ZNF2D | P*_CTR4-2-_ZNF2* (JEC21) *NEO^r^ Kan^R^* | This study |
| pXG-ZNF2A | P*_GAL10-_ZNF2* (H99) *NEO^r^ Kan^R^* | This study |
| pXG-ZNF2D | P*_GAL10-_ZNF2* (JEC21) *NEO^r^ Kan^R^* | This study |
| pXL1-MAT2D | P*_GPD1-_MAT2* (JEC21) *NEO^r^ Kan^R^* | This study |
| pXL1-CFL1 | P*_GPD1-_CFL1* (H99) *NEO^r^ Kan^R^* | This study |
| pXL1-00596 | P*_GPD1-_*CNAG_00596 *NEO^r^ Kan^R^* | This study |
| pXC-00925 | P*_CTR4-2-_* CNAG_00925 *NEO^r^ Kan^R^* | This study |
| pXC-01121 | P*_CTR4-2-_* CNAG_01121 *NEO^r^ Kan^R^* | This study |
| pXL1-05729 | P*_GPD1-_* CNAG_05729 *NEO^r^ Kan^R^* | This study |
| pXL1-05778 | P*_GPD1-_* CNAG_05778 *NEO^r^ Kan^R^* | This study |
| pXC-06239 | P*_CTR4-2-_* CNAG_06239 *NEO^r^ Kan^R^* | This study |
| pXL1-06411 | P*_GPD1-_* CNAG_06411 *NEO^r^ Kan^R^* | This study |
| pXL1-07422 | P*_GPD1-_* CNAG_07422 *NEO^r^ Kan^R^* | This study |
| pLKB25 | *mCherry NEO Amp^R^* | [3] |
| pXL1-NCfl1A::mCherry | P*_CFL1-_* *CFL1 (H99)::mCherry* *NEO^r^ Kan^R^* | This study |
| pXL1-NCfl1D::mCherry | P*_CFL1-_* *CFL1 (JEC21)::mCherry* *NEO^r^ Kan^R^* | This study |
| pXL1-Cfl1::mCherry | P*_GPD1-_* *CFL1::mCherry* *NAT Kan^R^* | This study |
| pXL1-GZNF2D | P*_GAL10-_* *ZNF2* *(JEC21)* *NEO^r^ Kan^R^* | This study |
| pXC-cfl1(sigPΔ)::mCherry | P*_CTR4-2-_* *CFL1(sigP*Δ*)::mCherry* *NEO^r^ Kan^R^* | This study |
| pXC-Cfl1::mCherry | P*_CTR4-2-_* *CFL1::mCherry* *NEO^r^ Kan^R^* | This study |

1. Hsueh YP, Xue C, Heitman J (2009) A constitutively active GPCR governs

morphogenic transitions in *Cryptococcus neoformans*. EMBO J 28: 1220-1233.

2. Chayakulkeeree M, Rude TH, Toffaletti DL, Perfect JR (2007) Fatty acid synthesis

is essential for survival of *Cryptococcus neoformans* and a potential fungicidal

target. Antimicrob Agents Chemother 51: 3537-3545.

3. Kozubowski L, Heitman J (2010) Septins enforce morphogenetic events during

sexual reproduction and contribute to virulence of *Cryptococcus neoformans*. Mol

Microbiol 75: 658-675.
