# Supplementary material for: The Link between Morphotype Transition and Virulence in Cryptococcus neoformans
Source: PLoS Pathog. 2012 Jun 21;8(6):e1002765. doi: 10.1371/journal.ppat.1002765 (PMC3380952; doi:10.1371/journal.ppat.1002765)
Supplement: Table S3 — Primers used in this study. (DOC) [file ppat.1002765.s016.doc]

Table S3. Primers used in this study.

| **Comments** | **Primer name** | **Sequence** |
| --- | --- | --- |
| *ZNF2* (H99) overexpression | Linlab 599 | GTTTAATTAACCAGTCGATAACTCGATTTAG |
|  | Linlab 600 | TGATCTGGCCGGCCCGGCCTGATATGCTGGAG |
| *ZNF2* (JEC21) overexpression | Linlab 602 | GTTTAATTAAAGGACCAGTCAATACTTTGGA |
|  | Linlab 603 | TGATCTGGCCGGCCAATCAAAATGGACGTTCTTGA |
| P*_CTR4-2_* | Linlab677 | AAGGAAAAAAGCGGCCGCGCCAAATCAAGCTTATCGATG |
|  | Linlab678 | TGATCTGGCCGGCCGATTGGTGAAGTCGTTGTCGTA |
| P*_GAL10_* | Linlab646 | AAGGAAAAAAGCGGCCGCGCGGGGAGTACAGGCTAAGCGT |
|  | Linlab647 | TGATCTGGCCGGCCGGTAACTCGAGTCTGTTCAAAAACCA |
| *MAT2*  overexpression | Linlab767 | TGATCTGGCCGGCCTCACATCATGGCGGCCAATCA |
|  | Linlab768 | GTTTAATTAAACTAGGGTATACCACTCAGGCGTCT |
| *CFL1* (H99)  overexpression | Linlab827 | TGATCTGGCCGGCCAGACATCATGCTCGTTAGCAACATC |
|  | Linlab828 | GTTTAATTAAGAACGCTCAAGAGGAATATTCAGAC |
| CNAG_05729  overexpression | LinLab759 | TGATCTGGCCGGCCCATCAAGATGCGATTCACCTCCAT |
|  | LinLab760 | GTTTAATTAATGATAACGGGGCTTAAAGAATAGA |
| CNAG_06411  overexpression | LinLab755 | TGATCTGGCCGGCCTAAAATAATGTTCCCTTTCAACTCT |
|  | LinLab756 | GTTTAATTAAGTGATTCCTTTAGCAAGTAATAGAA |
| CNAG_06239  Overexpression | LinLab825 | TGATCTGGCCGGCCTCCCATCATGTCTGCGTCAATT |
|  | LinLab826 | GTTTAATTAAATGTATCATTATCCATCTATGGCAA |
| CNAG_07422 overexpression | Linlab761 | TGATCTGGCCGGCCACCCGCCATGTTCTCGTCCACTA |
|  | Linlab762 | GTTTAATTAACGTCGATATACATCTTACAGCTGGA |
| CNAG_05778  overexpression | Linlab831 | TGATCTGGCCGGCCGCCCACTATGCCCGCTAACTTTA |
|  | Linlab832 | GTTTAATTAATTGGTTAAAATGATACCTAAAATTGTC |
| CNAG_00596  overexpression | Linlab765 | TGATCTGGCCGGCCCAACACGATGATACGCCTCAACATC |
|  | Linlab766 | GTTTAATTAACCATCGGACATAGAAGATTAGATGA |
| CNAG_00925  overexpression | Linlab763 | TGATCTGGCCGGCCTGTCAGGATGCGTTTTACTTCTATC |
|  | Linlab764 | GTTTAATTAATTGCTGGTTCCAGTACTTAAAGGAT |
| CNAG_01121  overexpression | Linlab833 | TGATCTGGCCGGCCCAGCAACATGTTCTTCACATATCTC |
|  | Linlab834 | GTTTAATTAACAAAAAGAGAAGAATTATCTGCTGC |
| *MAT2* (JEC21)  overexpression | Linlab767 | TGATCTGGCCGGCCTCACATCATGGCGGCCAATCA |
|  | Linlab768 | GTTTAATTAAACTAGGGTATACCACTCAGGCGTCT |
| *cfl1* deletion (JEC21/20 and XL280) | 16025 | CCGGCCGAACATCTACG |
|  | 16026 | CTGGCCGTCGTTTTACCGAGCATGCCGTCCACAG |
|  | 16027 | GTCATAGCTGTTTCCTGGGCACCCAGTGTATCGCTG |
|  | 16028 | GGTGCGTGCCAAAGGAC |
|  | 16029 | CGATCGAGGGCAGGGAAG |
|  | Linlab866 | GCCCTTGCTCACCATAACAGCGATACACTGGGTGCCAT |
|  | Linlab867 | ATGGCACCCAGTGTATCGCTGTTATGGTGAGCAAGGGC |
|  | Linlab864 | GTTTAATTAATTACTTGTACAGCTCGTCCA |
| P*_CFL1_-CFL1-mCherry* (H99) | Linlab861 | TCAGCGGCCGCATAGAAATGCCCGTTGGCATT |
|  | Linlab862 | GCCCTTGCTCACCATGACAGCGATACATTCGGTGCCA |
|  | Linlab863 | TGGCACCGAATGTATCGCTGTCATGGTGAGCAAGGGC |
|  | Linlab864 | GTTTAATTAATTACTTGTACAGCTCGTCCA |
| P*_CFL1_-CFL1(sigP*Δ*)-mCherry* (H99) | Linlab948 | TGATCTGGCCGGCCAGACATCATGAACGGAATCTCCTTG  GCGATG |
|  | Linlab862 | GCCCTTGCTCACCATGACAGCGATACATTCGGTGCCA |
|  | Linlab863 | TGGCACCGAATGTATCGCTGTCATGGTGAGCAAGGGC |
|  | Linlab864 | GTTTAATTAATTACTTGTACAGCTCGTCCA |
| P*_CFL1_-CFL1-mCherry* (JEC21/20) | Linlab865 | TCAGCGGCCGCCGAACATCTACGAGAGCCATCA |
|  | Linlab866 | GCCCTTGCTCACCATAACAGCGATACACTGGGTGCCAT |
|  | Linlab867 | ATGGCACCCAGTGTATCGCTGTTATGGTGAGCAAGGGC |
|  | Linlab864 | GTTTAATTAATTACTTGTACAGCTCGTCCA |
| *TEF1* QPCR | Linlab 329 | CGTCACCACTGAAGTCAAGT |
|  | Linlab 330 | AGAAGCAGCCTCCATAGG |
| *MAT2* (CNAG_06203) QPCR | Linlab 975 | GCTCCTCGCTACATCTCCTCA |
|  | Linlab 976 | TGTTTCGGTCTACGATACCAGTT |
| *STE3* (CNAG_06808) QPCR | Linlab 977 | CCCTGCATCATCCATCCTTT |
|  | Linlab 978 | GTATTCCCCCATCGCATCAA |
| *STE6* (CNAG_03600) QPCR | Linlab 973 | TAGTTTGAGGGAAGCGGATGT |
|  | Linlab 974 | GACCCGTAGGCGATGTTTTC |
|  | Linlab 732 | TCATCTCGGACCTCTTCTG |
| *MF1* (CNAG_07406) QPCR | Linlab 577 | GCCTTCACTGCCATCTTC |
|  | Linlab 578 | TTAGGCGATGACGCATAG |
| *ZNF2* (CNAG_03366) QPCR | Linlab 333 | GCTCAACGTCACATCAAGC |
|  | Linlab 334 | CTTGTGGCTCGTCAAAATG |
|  | LinLab330 | AGAAGCAGCCTCCATAGG |
| *ACT1* QPCR | LinLab323 | GCCCTTGCTCCTTCTTCTAT |
|  | LinLab324 | GACGATTGAGGGACCAGACT |
|  | Linlab820 | GCAACCACCACAAGAGTTC |
| CNAG_04944 | Linlab803 | GGTGAAGAATCTCCTGAAGG |
|  | Linlab804 | CATAGCAAGCAACCCAGTC |
| CNAG_05729 QPCR | LinLab347 | CTGTCCAACGTTATCCAGAC |
|  | LinLab348 | CCACCAAGGAGTGACTTTAG |
| CNAG_06411  QPCR | LinLab345 | CAGATGATTGAAGTGCCTATG |
|  | LinLab346 | CCAGAGTGGTAGTTGAAGTTG |
| CNAG_06239 QPCR | LinLab343 | CACTCACTCGACGATCCTG |
|  | LinLab344 | CAAGGACCCTTGACAGGAC |
| CNAG_07422 QPCR | Linlab731 | TCTTGTGGTGACGAAACTGT |
|  | Linlab732 | TCATCTCGGACCTCTTCTG |
| CNAG_05778 QPCR | Linlab799 | CTTCAGTGCTGCTGTGAGTG |
|  | Linlab800 | AGTGACGCTTGAAGCAGTAG |
| CNAG_00596 QPCR | Linlab718 | GTGGCTACATCGATTGGAC |
|  | Linlab719 | AGAGTTGACGCATTGGTAGT |
| CNAG_00925 QPCR | Linlab733 | CAGGCAATTCATCAACATTC |
|  | Linlab734 | CACCAACATCAATGAGAAGG |
| *CFL1* QPCR | Linlab795 | GGTCTCTCCATGCTTGTACC |
|  | Linlab796 | CCAGATTTGCAGCTGTAGAC |
| CNAG_01121  QPCR | Linlab801 | GGTCTACTACATCCCACAATG |
|  | Linlab802 | CCTGTGAGGTCGTACCAGT |
